# Contrasting Phylogeography of Sandy vs. Rocky Supralittoral Isopods in the Megadiverse and Geologically Dynamic Gulf of California and Adjacent Areas

**DOI:** 10.1371/journal.pone.0067827

**Published:** 2013-07-02

**Authors:** Luis A. Hurtado, Eun Jung Lee, Mariana Mateos

**Affiliations:** Department of Wildlife and Fisheries Sciences, Texas A&M University, College Station, Texas, United States of America; University of Poitiers, France

## Abstract

Phylogeographic studies of animals with low vagility and restricted to patchy habitats of the supralittoral zone, can uncover unknown diversity and shed light on processes that shaped evolution along a continent’s edge. The Pacific coast between southern California and central Mexico, including the megadiverse Gulf of California, offers a remarkable setting to study biological diversification in the supralittoral. A complex geological history coupled with cyclical fluctuations in temperature and sea level provided ample opportunities for diversification of supralittoral organisms. Indeed, a previous phylogeographic study of *Ligia*, a supralittoral isopod that has limited dispersal abilities and is restricted to rocky patches, revealed high levels of morphologically cryptic diversity. Herein, we examined phylogeographic patterns of *Tylos*, another supralittoral isopod with limited dispersal potential, but whose habitat (i.e., sandy shores) appears to be more extensive and connected than that of *Ligia*. We conducted Maximum Likelihood and Bayesian phylogenetic analyses on mitochondrial and nuclear DNA sequences. These analyses revealed multiple highly divergent lineages with discrete regional distributions, despite the recognition of a single valid species for this region. A traditional species-diagnostic morphological trait distinguished several of these lineages. The phylogeographic patterns of *Tylos* inside the Gulf of California show a deep and complex history. In contrast, patterns along the Pacific region between southern California and the Baja Peninsula indicate a recent range expansion, probably postglacial and related to changes in sea surface temperature (SST). In general, the phylogeographic patterns of *Tylos* differed from those of *Ligia*. Differences in the extension and connectivity of the habitats occupied by *Tylos* and *Ligia* may account for the different degrees of population isolation experienced by these two isopods and their contrasting phylogeographic patterns. Identification of divergent lineages of *Tylos* in the study area is important for conservation, as some populations are threatened by human activities.

## Introduction

The dynamic interaction between sea and land can greatly enhance biological diversification at the ocean supralittoral or splash zone. This zone comprises a narrow (few meters) vertical stretch of the shoreline; yet, it spans an extensive region at a regional and global scale [1]. A few animals have adapted to live exclusively in the supralittoral, despite the predominantly harsh conditions that characterize this zone. These include regular exposure to extreme temperatures, to air, to fresh water from rain, to seawater from wave splash and storm surge, and to predation by land animals and seabirds [1]–[3]. Diverse patchy habitats occur in the supralittoral (e.g. rocky vs. sandy), resembling ‘islands’ along the vast coastline [4]. High levels of population isolation are expected in animals whose entire life cycle is restricted to a single patchy habitat type, because surrounding unsuitable habitats can constitute effective dispersal barriers [4]–[6]. In addition, tectonic activity, eustatic sea level changes, erosion, hurricanes, and sediment input from rivers, can dramatically modify the distribution of coastline habitats [7], thereby influencing the evolutionary histories of supralittoral endemics. Examination of molecular phylogenetic patterns of highly isolated non-vagile animals that are restricted to specific supralittoral patchy habitats can help to understand: (1) biogeographic processes that occurred along the coastline of a region; (2) whether biological diversification processes have been common across faunas from different patchy habitats (e.g. sandy vs. rocky shores); and (3) their levels of morphologically cryptic diversity, which is relevant to taxonomy, and to the protection of the unique biodiversity found in an environment subject to high pressure from human disturbances [3].

The Pacific region between southern California and central Mexico, including the Gulf of California, provides a remarkable setting to study biological diversification in the supralittoral zone. A complex and controversial geological history, extending back to the Miocene, coupled with the Quaternary’s cyclical pattern of rising and falling global temperatures and transgressing and regressing seas [8]–[10], provided ample opportunities for range expansions/contractions, vicariance and allopatric genetic differentiation of supralittoral organisms in this region [6], [11]–[15]. The origin of the Gulf of California basin itself dates back to at least 12 Ma [16]; and the formation of this basin and the Baja California Peninsula encompassed a complex geological process [17]. The dynamic geological history of this region appears to have played an important role in the diversification of supralittoral isopods [6]; and is considered an important driver of the extraordinarily high marine and terrestrial biodiversity in this region [6], [18]–[23]. The Gulf of California harbors >6,000 nominal marine animal species and subspecies, of which ∼5,000 are marine invertebrates (∼16% endemic), but actual diversity may be substantially greater [24]. In addition, elevated levels of cryptic diversity may be common within some groups and habitats, such as supralittoral isopods [6].

A phylogeographic study found high levels of allopatric genetic differentiation among populations of supralittoral rocky intertidal isopods of the genus *Ligia*, Fabricius 1798 in the Pacific region between central Mexico and southern California, including the Gulf of California [6]. Genetic divergences among numerous lineages of *Ligia* are very large [Kimura-2-parameter (K2P) >10–>20% for the Cytochrome Oxidase I gene (COI)], indicating long-standing isolation of populations, and suggesting the presence of a complex of cryptic species; even though only one native species, *Ligia occidentalis*, is traditionally recognized in this region. The high level of cryptic biodiversity found in *Ligia* implies that the diversity of marine isopods in the Gulf is greater than the current 82 nominal species recorded [24]. The high allopatric diversity found in *Ligia* is consistent with the biological characteristics of this isopod: (1) direct development (common to all peracarids); and (2) high specificity to the patchy supralittoral rocky intertidal habitat. This isopod actively avoids entering the sea, although it retains the ability for underwater gas exchange and can swim over short distances (i.e., few meters). In addition, it does not venture into terrestrial environments adjacent to its patchy rocky intertidal habitat (e.g. large stretches of sandy beaches), probably due to its extremely low desiccation tolerance and high predation risk [2].

Another supralittoral endemic isopod expected to exhibit high levels of cryptic diversity and to retain phylogeographic signatures of past events in this region is *Tylos* Audouin 1826, a genus found worldwide mainly in tropical and subtropical shores [5]. Ocean dispersal by members of *Tylos* is limited, because they are also direct developers, are unable to swim, and survive at most few hours under water [5], [25], [26]. It is speculated, however, that juveniles of certain *Tylos* species may be able to surf by rolling themselves into a ball, which may facilitate over-water dispersal among nearby beaches [5], [25]–[27]. Long distance terrestrial dispersal is also limited. During the day, these isopods remain inactive and buried in the sand near the previous high tide mark; which protects them from high temperatures, desiccation, predation, and dislodgement by waves [3], [28], [29]. At night, they emerge to the intertidal portion of the sand that is not submerged, where they forage on detritus and algae. Subtropical populations of *Tylos*, such as in southern California, remain buried and inactive during the winter [26], [27], [30]. *Tylos* is commonly found in sandy beaches of the Pacific region between central Mexico and southern California, including the Gulf of California. At some locations within this region, *Tylos* co-occurs with *Ligia*, where it can be found buried in the sand beneath supralittoral rocks or in the sand adjacent to rocky supralittoral patches. The extent and connectivity of the habitat occupied by *Tylos* in this region is greater than that of *Ligia*. Examination of the phylogeographic patterns of *Tylos* will provide further insights into the processes that led to diversification in the supralittoral zone of this megadiverse and geologically dynamic region.

Herein, we studied the phylogeographic patterns of the sandy beach supralittoral endemic *Tylos* in the region between southern California and central Mexico, including the Gulf of California. We examined DNA sequences from mitochondrial and nuclear markers, as well as the shape of the ventral plates of the fifth pleonite, a species-diagnostic morphological character used in *Tylos*. As expected from its biological characteristics, we discovered high levels of cryptic diversity within this isopod. This information is very relevant, because *Tylos* populations in the study area are highly threatened by coastal human activities [31]–[33]. We discuss the taxonomic implications of our molecular and morphological analyses. We interpret the phylogeographic patterns of *Tylos* in relation to past tectonic and climatic events, and compare them with those of the rocky supralittoral isopod *Ligia*. The general phylogeographic patterns of *Tylos* and *Ligia* were very different. This is surprising because the two isopods share similar geographic distributions and dispersal limitations, and are thus, expected to have been exposed to many of the same past events that impacted the supralittoral in the study area (i.e., glaciations, sea level fluctuations, and vicariant events related to the formation of the Gulf of California and Baja California Peninsula). Differences in the extension and connectivity of the two different habitats they occupy (i.e., sandy vs. rocky) may account for the different degrees of population isolation experienced by the two isopods and their contrasting phylogeographic patterns.

## Materials and Methods

### 2.1 Sampling

**Figure 1:**
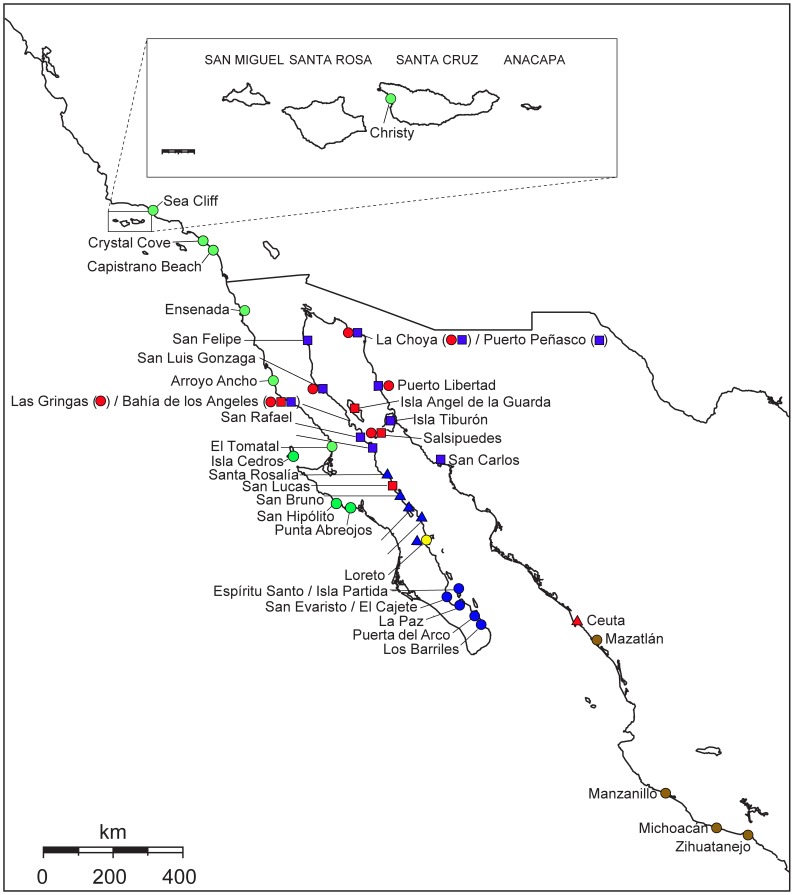
Sampled localities in the study area. Colors and shapes correspond to clades in Fig. 2. * denotes location of Guerrero Negro Lagoon in the central Baja California Peninsula.

We collected *Tylos* specimens from 45 rocky and sandy beach localities between central Mexico and Southern California, including the Gulf of California (Fig. 1). Most of the samples were collected during 2008–2011. Collection information for the samples is shown in Table S1. All necessary permits were obtained for the described study, which complied with all relevant regulations. Collections were conducted under scientific collecting permits: California Department of Fish and Game (USA) No. 9881; and Comisión Nacional de Acuacultura y Pesca (Mexico) No. DGOPA.l0337.020908.2952.

### 2.2 Molecular Methods

Genomic DNA was isolated from 2–4 legs per specimen with the DNEasy kit, following the manufacturer’s protocol (Qiagen, Inc., Valencia, CA). For 1–9 individuals per locality, we PCR-amplified and sequenced one or two mitochondrial (mt) gene fragments (16S rDNA and COI; see Table S1). From this dataset (Dataset S1), we selected at least one individual per locality per distinct lineage (see Figure S1) to examine an additional four mitochondrial and two nuclear markers. Primer sequences and annealing temperatures are given in Table S2. The amplified mitochondrial (mt) segments were: 16S ribosomal (r)DNA (primers 16Sar and 16Sbr from [34]); 12S rDNA (primers 12S-CRF and 12S-CRR from [35] and Crust-12f and Crust-12r from [36]); Cytochrome Oxidase I gene (COI; primers HCO-2198 and LCO-1490 from [37] and newly designed primers reported in Table S2); Cytochrome b gene (Cytb; primers Cytb-151F, Cytb-144F, Cytb-270R, and Cytb-272R from [38]); and a ND6/ND4 segment that includes portions of the 16S rDNA (non-overlapping with the aforementioned segment), NADH4, NADH6 genes, and intervening tRNAs (primers N4 and 16S2 from [39]). In addition, the nuclear genes examined were the highly variable V4 region of the 18S rDNA gene (primers 18S-3F and 18S-5R from [40]); and the Histone 3 (H3A) gene (primers H3-aF and H3-aR [41]). PCR-amplified products were cleaned with Exonuclease and Shrimp Alkaline Phosphatase, and subsequently cycle sequenced at the University of Arizona Genetics Core. We used Sequencher 4.8 (Gene Codes, Ann Arbor, MI) for sequence editing and primer removal. None of the protein-coding sequences had premature stop codons or frame shifts, suggesting that they are not pseudogenes. All sequences have been deposited under GenBank Accession Numbers KF007342–KF007569 and KF007571–KF007889.

### 2.3 Sequence Alignment and Datasets

Non-protein-coding sequences were aligned with MAFFT v.6.0 [42], as implemented in http://mafft.cbrc.jp/alignment/server/, with the Q-INS-I strategy, which considers secondary structure of RNA, and with the L-INS-i strategy with default parameters (e.g. Gap Opening penalty = 1.53). Resulting alignments were edited manually within MacClade v.4.06 [43]. Regions for which homology could not be confidently established were identified with GBlocks v.0.91b [44], and excluded from the phylogenetic analyses. The following GBlocks parameters were used: “Allowed Gap Positions” = half; “Minimum Length Of A Block” = 5 or 10; and “Maximum Number Of Contiguous Nonconserved Positions” = 4 or 8. Alignments showing included and excluded positions are available in Dataset S2.

As outgroups, we used two lineages from the Caribbean (*Tylos* sp. from Yaguanabo, Cuba and *T. niveus*, from Puerto Rico), which according to phylogenetic analyses of most of the species in the genus *Tylos* [45], are the closest relatives of the study area lineages. Phylogenetic analyses were conducted on the following datasets (see Table S3): (1) concatenated mitochondrial loci (MT); (2) the nuclear 18S rDNA; (3) the nuclear H3A; and (4) concatenated mitochondrial and nuclear loci (MT+NC).

### 2.4 Phylogenetic Analyses

To determine the most appropriate model of DNA substitution among 88 candidate models on a fixed BioNJ-JC tree, we used jModeltest v0.1.1 [46] under the Akaike Information Criterion (AIC), corrected AIC(c), and Bayesian Information Criterion (BIC) (Table S3). We used the closest more complex model available in the corresponding ML and Bayesian analyses (Table S4), except that when a proportion of invariable sites (I) and a Gamma distribution of rates among sites (G) was selected according to jModeltest, we excluded parameter I to avoid problems related to dependency between two parameters (see RaxML manual and [47]). In addition, to assess robustness of the results to substitution model, we also used the complex model GTR+G. Several data partitioning schemes were implemented, including: (a) all positions within a single partition; (b) the best partitioning scheme according to the BIC implemented in PartitionFinder v.1.0 [48]; and (c) 1–3, partitions not specified a priori (i.e., BayesPhylogenies; Table S4). The following parameters were used in PartitionFinder: branch lengths = linked; models = all; model selection = BIC; search = greedy; and a priori partitioning by a combination of each gene and codon position.

For the ML analyses, two approaches were employed: (a) a Rapid Bootstrap followed by ML search in RaxMLGUI v.1.0, which includes the executable files of RAxML v.7.3.0 [49], [50]; and (b) GARLI v.2.0 [51], which uses genetic algorithms for the ML search. Clade support was examined by non-parametric bootstrap analyses (100–1000 replicates) summarized with 50% majority rule consensus trees by the SumTrees script implemented in DendroPy v.3.10.1 [52].

For the Bayesian analyses, three programs were used. The first one was MrBayes v.3.2.1 [53]–[55], but such analyses have been reported to return high clade posterior probabilities in certain cases of known polytomies (a.k.a., the “star-tree paradox”) [56]. Therefore, we also applied one of the proposed strategies to alleviate this problem; i.e., the polytomy prior [57] as implemented in Phycas v.1.2.0 [58]. Finally, we used BayesPhylogenies v.1.1 to fit more than one substitution model to different positions in the dataset without the need for identifying the data partitions a priori [59]. Analyses of 1–3 partitions (i.e., patterns) were conducted.

The following criteria were used to evaluate convergence and adequate sampling of the posterior distribution: (a) Stable posterior probability values; (b) a high correlation between the split frequencies of independent runs as implemented in AWTY [60]; (c) small and stable average standard deviation of the split frequencies of independent runs; (d) Potential Scale Reduction Factor close to 1; and (e) an Effective Sample Size (ESS) >200 for the posterior probabilities, as evaluated in Tracer v.1.5 [61]. Samples prior to reaching a stationary posterior distribution were discarded (i.e., “burnin”; Table S4).

Pairwise genetic distances with Kimura-2-parameter (K2P) correction were estimated with MEGA v.5 [62] for the COI gene alone and for the remaining mitochondrial genes combined. Ambiguous positions were ignored for each sequence pair comparison.

### 2.5 Examination of Pleon Ventral Shapes

The shape of the ventral plates of the fifth pleonite is commonly used as a species-diagnostic character for *Tylos* [63]. We examined this trait in individuals from the study area that belonged to genetically differentiated lineages, as indicated by the phylogenetic results. Specimens were photographed under a dissecting microscope. We visually compared this trait among lineages from the study area, *T. punctatus* syntype specimens, and most of the other known species in the genus.

## Results

### 3.1 Phylogenetic Relationships of *Tylos* within the Study Area

**Figure 2:**
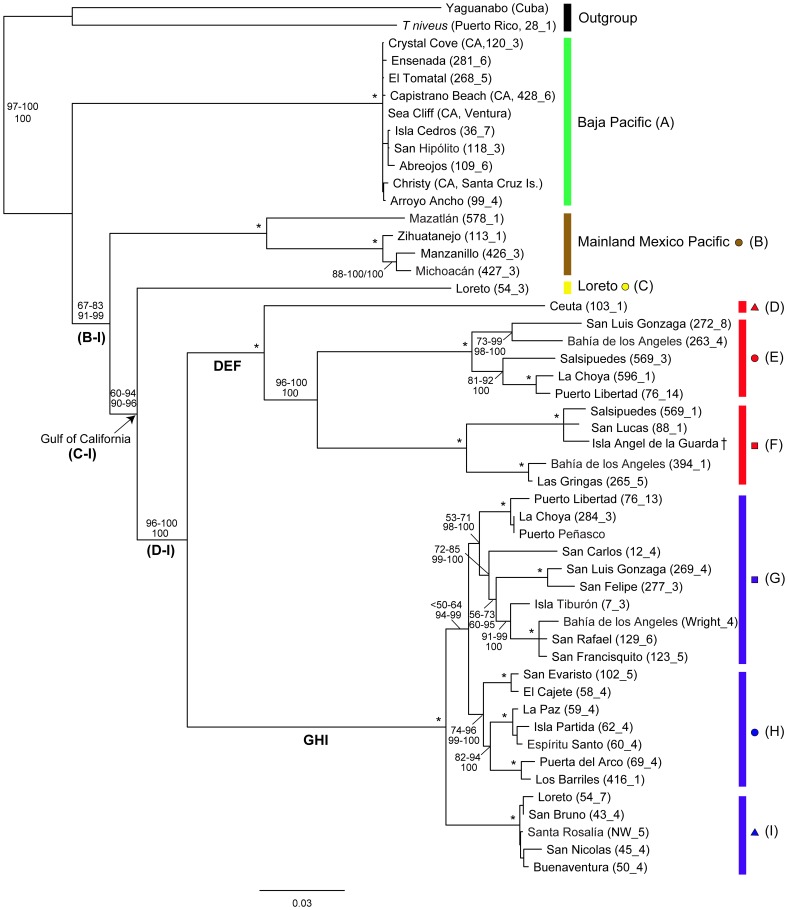
Inferred phylogeny of *Tylos* in the study area, based on the concatenated mitochondrial+nuclear loci. Majority-rule consensus tree (RaxML bootstrap). Colors and shapes correspond to clades in Fig. 1. Numbers by nodes indicate the corresponding range of Bootstrap Support (BS; top or left) for Maximum likelihood (RaxML, Garli, PartitionFinder); and Posterior Probabilities (PP; bottom or right) for Bayesian inference methods (MrBayes, Phycas, BayesPhylogenies), including all partitioning schemes. * denotes nodes that received 100% support for all methods. Nodes receiving less than 50% support for all methods were collapsed and denoted with <50. †: relationship based on 16S sequence only: Isla Angel de la Guarda.

The concatenated mitochondrial dataset (MT) of the study area included 50 taxa and 2992 characters, of which 1058 were parsimony informative (Table S3). The nuclear H3A dataset included 34 taxa and 285 characters, of which 20 were parsimony informative. The nuclear 18S rDNA dataset included 40 taxa and 520 characters, of which 110 were parsimony informative. Finally, the combined mitochondrial and nuclear (MT+NC) dataset included 50 taxa and 3797 characters, of which 1188 were parsimony informative. Alignments are shown in Dataset S2. Figure 2 depicts the inferred phylogenetic relationships based on the MT+NC dataset among the samples of *Tylos* from the study area. Phylogenetic reconstructions based on the mitochondrial-only and individual nuclear genes, which are presented in Figures S2–S4 (parameters for analyses in Tables S5–S6), were generally congruent, with two exceptions addressed below. The MT+NC analyses revealed five main monophyletic lineages (*A*, *B*, *C*, *DEF*, and *GHI*; identified by different colors in Figs. 1 and 2) that were separated by ∼10–19% COI K2P divergence (Table S7). The first main lineage (*Clade A*; green in Figs. 1 and 2), supported by 100 PP and BS values, included all Pacific samples between the Baja California Peninsula and southern California. This clade was characterized by very shallow divergences (≤0.6% COI K2P; Table S7). The second main lineage (*Clade B*; brown in Figs. 1 and 2), supported by 100 PP and BS values, included all the samples collected in Mexico between Mazatlan, at the southern mainland limit of the Gulf of California, and Zihuatanejo, in southern Mexico. In this clade, a deep divergence (∼10% COI K2P; Table S7) was observed between the sample from Mazatlan (B–I in Table S7) and the other localities (B–II in Table S7), which differed from each other by <2% COI K2P. The third main lineage (*Lineage C*; yellow in Figs. 1 and 2) was found only at the locality of Loreto, in the central Gulf portion of the Baja California Peninsula. The fourth main lineage (*Clade DEF*; red in Figs. 1 and 2), supported by 100 PP and BS values, was divided into three lineages (*D*, *E*, and *F*) that differed from each other by ∼11–14.5% COI K2P (Table S7). The fifth main lineage (*Clade GHI*; blue in Figs. 1 and 2), supported by 100 PP and BS values, was also divided into three main clades (*G*, *H*, and *I*) that differed from each other by ∼3.5–7% COI K2P (Table S7).

Phylogenetic relationships among the five main lineages were relatively well resolved in the MT+NC concatenated dataset (Figure 2), but two major discrepancies were observed among the individual datasets (MT vs. H3A vs. 18S rDNA). First, most of the analyses of the MT dataset placed *Lineage C* as sister to a clade formed by *Clades D–I* (Figure S2). In contrast, analyses of the 18S rDNA gene placed *Lineage C* as sister to *Clade DEF* (Figure S3), whereas analyses of the H3A gene placed it in a group with members of the *Clade GHI* (Figure S4; supported by a single unambiguous third-codon position change; not shown). Analyses of the concatenated MT+NC dataset (Figure 2) recovered the same relationship as the MT dataset alone, but with higher clade support. A second notable discrepancy among markers was observed. Analyses of the H3A gene alone recovered a sister relationship between *Clade A* and *Clade DEF*. This relationship however, was only supported by two unambiguous changes at third-codon positions. Analyses of the 18S rDNA gene were unable to resolve this relationship, and analyses of the MT and the MT+NC dataset recovered the sister relationships of *Clades DEF* and *GHI* with high clade support. Although we acknowledge that multispecies coalescent analyses of additional unlinked nuclear markers are likely needed to resolve relationships with more certainty [64], the relationships inferred with the MT+NC concatenated dataset are at this point the most plausible hypothesis.

The MT+NC analyses suggest that the earliest divergence occurred between *Clade A* (green; Pacific localities between southern California and Baja California) and the remaining lineages *B–I* (Fig. 2). The monophyly of the *Clade B–I* obtained 91–99 PP and 67–83 BS. Within this clade, the earliest divergence occurred between *Clade B* (brown; Pacific localities between Mazatlan and Zihuatanejo) and the clade containing all of the Gulf lineages (*C–I*). Support for the monophyly of the Gulf clade (*C–I*) was 90–96 PP and 60–94 BS. Within the Gulf lineages, the earliest divergence occurred between *Lineage C* (yellow; from Loreto), and the other lineages (*D–I*). Support for the monophyly of *Clade D–I* was 100 PP and 96–100 BS. Within this group, *Clades DEF* (red) and *GHI* (blue) were reciprocally monophyletic with 100% support in all analyses.

Within *Clade DEF*, several distinct and divergent lineages were identified. This clade was divided into three main lineages: two reciprocally monophyletic clades distributed in the northern Gulf (*E* and *F*; red circles and squares, respectively) that differed by ∼11–13% COI K2P (Table S7); and their sister lineage (*D*; red triangle), found only at Ceuta (∼70 Km north of Mazatlan) in the mainland southern Gulf, and divergent from *E* and *F* by ∼11–14.5% COI K2P (Table S7). *Clade E* was divided into two reciprocally monophyletic groups divergent by ∼7.7% COI K2P: one was found in the northern Gulf Baja localities of San Luis Gonzaga and Bahia de los Angeles; the other was found in the northern Gulf mainland localities of Puerto Libertad and La Choya, and the midriff island of Salsipuedes off northern Gulf Baja. *Clade F* was divided into two reciprocally monophyletic groups divergent by ∼6.9% COI K2P: one was found in Bahia de los Angeles; the other was found in the midriff islands of Angel de la Guarda and Salsipuedes, and in the central Gulf Baja locality of San Lucas.

Several differentiated lineages were observed in *Clade GHI*. This clade was divided into three main lineages distributed allopatrically: *Clade G* (blue squares) distributed in the northern Gulf of California; *Clade H* (blue circles) distributed in the Baja California Cape Region; and *Clade I* (blue triangles) distributed in the central Gulf portion of the Baja California Peninsula. Divergence among *Clades G*, *H*, and *I* ranged between ∼3.5–7% COI K2P (Table S7). A closer relationship between *Clades G* and *H* was suggested by Bayesian analyses with 94–99 PP (<50–64 BS). Divergences within *Clade G* were as high as ∼6% COI K2P (Table S7). In this clade, the localities from Puerto Libertad, Puerto Peñasco, and La Choya formed a differentiated group. Samples from San Luis Gonzaga and San Felipe formed another differentiated group. Bahia de los Angeles, San Francisquito, and San Rafael corresponded to another clade, whose sister lineage was Isla Tiburon. The sample from San Carlos was highly divergent (3.1–6.1% COI K2P; not shown) from the others. Divergences within *Clade H* were as high as ∼4.6% COI K2P. Within *Clade I*, observed divergences were <0.6% COI K2P (Table S7).

### 3.3 Morphology of the Ventral Plates of the Fifth Pleonite

Differences in the shape of the ventral plates of the fifth pleonite were detected among some of the main *Tylos* lineages found in the study area (Figure S5). Individuals from *Clade A* (Pacific localities between Baja California Peninsula and southern California) can be easily distinguished from those of other clades. Their pleon ventral shape is highly similar to that of *T. punctatus* syntype specimens from San Diego, California, suggesting they correspond to this species (Figure S5). The ventral plates of the fifth pleonite of *Clade A* are characterized by a curvilinear upper edge, a narrowly rounded tip, and a distal part that is not much wider than the basal portion. In *Clade B* (Pacific localities between Mazatlan and Zihuatanejo), the distal part is much wider, has a straighter upper edge, and does not end in a narrow tip. *Clade C* (from Loreto) is more similar to *Clade B* than to *Clade A*, but the distal part has sharper corners. Morphology of the single specimen obtained from *Lineage D*, which was found in Ceuta, could not be examined because it was destroyed during DNA extraction. Individuals from clades *E*–*I* can be distinguished from clades *A*–*C*, but no obvious differences were found among them. In clades *E*–*I*, the external vertex of the distal part is sharper than in *Clade B*, and the internal vertices are more rounded than in *Clade C.*

## Discussion

### 4.1 Cryptic Diversity and Taxonomy of *Tylos* in the Study Area

The generally high levels of genetic differentiation observed among *Tylos* populations from the study area are consistent with expectations stemming from its limited vagility and the fragmented nature of its habitat. High levels of allopatric genetic differentiation are also reported for rocky intertidal supralittoral *Ligia* isopods in the study area [6]. These observations challenge earlier suggestions that littoral isopods are highly dispersive species [65], and sharply contrast with the lack of genetic structure observed throughout the Gulf of California in upper intertidal invertebrates that possess planktonic larvae (e.g. [66]).

The high divergences observed among multiple regional lineages (e.g. *A–I*) suggest that *Tylos* in the study area corresponds to several species, rather than one (or two), as currently recognized: *T. latreillii* Audouin 1826 and *T. punctatus* Holmes and Gay 1909. Mulaik [67] reports *T. latreillii* in the northern Pacific coast of the Baja California Peninsula and in the Gulf of California. This species however, was originally described from an unspecified location in Egypt [68], and currently lacks type specimens, rendering it a *nomen dubium* [69]. Given its type locality, *T. latreillii* likely corresponds to one of the species found in the Mediterranean Sea (*Tylos europaeus* or *Tylos ponticus*) [69], or in the Red Sea (*Tylos exiguus*) [70]. According to Taiti and Ferrara [70], the morphology of *T. latreillii* most closely resembles that of *T. exiguus*. Specimens from many localities around the world have been incorrectly assigned to *T. latreillii*, which has contributed to taxonomic confusion within the genus [29], [68], [70], [71]. Given that *T. latreilli* is unlikely to occur in our study area, *T. punctatus* appears to be the only valid described species in this region.

*Tylos punctatus* has been reported in southern California as far north as Santa Barbara, as well as along the Pacific coast of the Baja California Peninsula, the Gulf of California, and the Galapagos Islands [27], [30], [71], [72]. Several authors have suggested, however, that *T. punctatus* from southern California is taxonomically distinct from *Tylos* found in the Gulf of California [5], [26], [27], [30]. Our morphological and phylogenetic analyses indicate that *T. punctatus* is restricted to the Pacific region between southern California and the central Baja California Peninsula, corresponding to *Clade A* in our phylogeny. The type locality of *T. punctatus*, San Diego, California, is within the range of *Clade A*. In addition, the morphology of the ventral plates of the fifth pleonite of the *T. punctatus* syntype is highly similar to that of *Clade A* specimens, and distinct from the other lineages found in the study area. Furthermore, extremely reduced genetic divergences within *Clade A* (≤0.6%), suggest this lineage corresponds to a single species, which is highly divergent from the other lineages found in the study area. *Tylos insularis* from the Galapagos Islands is considered a synonymy of *T. punctatus* [71]. Nevertheless, the morphology of the ventral plates of the fifth pleonite of the Galapagos samples [73] is very different to that of *Clade A*, the *T. punctatus* syntype, and the other lineages from the study area. This, in addition to their geographic separation, indicates that *T. insularis* likely represents a distinct species, as suggested by Van Name (p. 414 in [74]).

The morphology of the ventral plates of the fifth pleonite is diagnostic for several, but not all of the divergent lineages identified in the study area. *Clades A*, *B*, and *C* are distinct from each other and from *Clades E–I* (*Lineage D* was not examined). *Clades E–I* share similar morphology despite high divergences. Thus, this character fails to consistently distinguish what appear to be cryptic species. Examination of other morphological characters may help to distinguish these separate lineages. Studies of marine invertebrates based on the same COI fragment examined in our study have found that intra-specific divergences are typically <3% [75]. Accordingly, *Clades A*, *B*, *C*, *D*, *E*, *F*, and *GHI*, which are highly differentiated from each other (∼11–17%), probably represent distinct species. Additionally, some of these clades may be comprised of additional species. For example, in *Clade B*, the sample from Mazatlan may correspond to one species, whereas the samples from Zihuatanejo, Manzanillo, and Michoacan to another (divergence of Mazatlan vs. the others is 9.3–9.9%; whereas divergence among the others is <2%). Similarly, divergences among *Clades G*, *H*, and *I* are 3.5–7%, and divergences >3% occur within *Clades E*, *F*, *G*, and *H*. Additional unknown lineages may occur in the Guaymas to Ceuta mainland portion of the Gulf, a region dominated by sandy beaches that we were unable to explore thoroughly.

### 4.2 Phylogeographic Patterns of *Tylos* in the Study Area

#### 4.2.1 Southern california-Baja california pacific clade (*Clade A*)

The multilocus shallow divergences of *Clade A* (e.g. ≤0.6% COI K2P) may be indicative of a recent drastic past bottleneck followed by a range expansion. Additional evidence consistent with population expansion for *Clade A* was observed in (a) sequence mismatch distribution analyses (i.e., *τ* >0; *θ_1_*> *θ_0_*; [76], [77]); (b) negative Tajima’s *D* (–1.2; albeit non-significant); and (c) negative and significant Fu's *Fs* (–4.4; *P*<0.003) (all analyses conducted in Arlequin v.3.5 [78] for the 16S rDNA+COI dataset). A recent expansion of *Clade A* may be explained by Pleistocene glacial and postglacial events. The mainly subtropical-tropical distribution of the genus *Tylos* [5] suggests that cold temperatures likely limit its distribution at upper latitudes. *Clade A* reaches the latitudinal upper limit of the genus along the northeastern Pacific. Temperatures in the Southern California Bight were drastically reduced during the Pleistocene glacial periods; sea surface temperature (SST) was 6–10°C lower than present at the last glacial maximum (∼18,500 ya) [79]. Therefore, populations of *Clade A* likely contracted and/or shifted south during glacial periods. In addition to shifts in temperature, glacial-interglacial cycles were associated with sea level changes (at the last glacial maximum, sea levels in the southern California Bight were ∼117 m below present [9]), and were accompanied by continuous fluctuations in distribution and size of sandy and rocky shores, which likely affected the habitat of *Tylos*. Indeed, the present-day sand-dominated coastlines of the Southern California Bight appear to have developed only over the past 4000–6000 years [80], likely providing recent opportunities for the dispersal of a sandy beach organism such as *Tylos*.

In the northern Channel Islands, *Tylos* is found only on the western coast of Santa Cruz Island and the eastern coast of Santa Rosa Island [33]. Isopods on Christy Beach, Santa Cruz Island, have the most common *Clade A* haplotype found at mainland localities, suggesting that colonization of the northern Channel Islands by *Tylos* was recent. It is possible that colonization occurred over land, when the four present-day Northern Channel Islands apparently formed a large contiguous land mass (∼17,000 years ago), whose eastern end may have been connected to the mainland [81]. Rafting and surfing have also been suggested as a potential mechanism for over-water dispersal in *Tylos* [5], [25]–[27]. Nevertheless, this may be a rather ineffective mechanism for dispersal, as *Tylos* isopods drown if submerged even for short periods [5].

The phylogeographic patterns of *Tylos* are remarkably distinct from those of *Ligia* in the Pacific region spanning southern California to the Baja California Peninsula. Whereas *Tylos* shows very shallow divergences (≤0.6% COI K2P) suggestive of a recent expansion, multiple highly divergent (>10–20% COI K2P) lineages of *Ligia* occur in this region [6]. These *Ligia* lineages are in turn sister to a highly divergent lineage (20–25% COI K2P) that is distributed from north of Point Conception to southern Oregon along the mainland, and in western cold-water localities of the Northern Channel Islands. The distribution limit between these two divergent *Ligia* lineages corresponds with the Point Conception biogeographic boundary [11]: a transition area between northern-cold and southern-warm water masses [82], [83]. Therefore, SST appears to be a major factor affecting the distribution of these two divergent lineages [11]. A drastic decrease in genetic divergences is observed among *Ligia* populations (<3.1% COI K2P) in their northern range between Point Conception and Oregon, which suggests a recent post-glacial expansion for this isopod north of the Point Conception biogeographic boundary [11]. Differences between *Ligia* and *Tylos* in their tolerance to low SST may explain the distinct latitudes at which each of these isopods exhibits signatures consistent with post-glacial expansions. Greater tolerance to lower SST may also explain the higher abundance of *Ligia* in the Northern Channel Islands, where it occurs at many localities on all islands spanning both cold and warmer SST [6], [11]; whereas *Tylos* appears to be restricted to localities with warmer SST. Although the observed association between SST and the distributions of *Ligia* and *Tylos* lineages (two essentially terrestrial organisms) may appear surprising, SST influences abiotic factors (e.g. air temperature, sea and land breezes, atmospheric humidity, and coastal fog [84]–[87]) likely relevant to their survival and reproduction [11]. In *Tylos*, these factors have been shown to affect daily and seasonal activity, beach zonation, reproduction, and geographic distribution [5], [30], [88].

Another striking contrast between *Ligia* and *Tylos* is observed along the Pacific coast of the Baja Peninsula. A deep phylogeographic break coincident with the Guerrero Negro Lagoon occurs in *Ligia* (12–15% COI K2P), indicating long-standing separation between populations on either side of the lagoon, which is probably associated with the formation of this lagoon and lack of rocky habitat [6]. The presence of this lagoon, however, does not appear to have impeded the recent expansion of *Tylos* in this region, where continuous sandy shores may have facilitated its dispersal.

#### 4.2.2 Gulf of California-Central Pacific Mexico clades

In contrast to the shallow pattern observed in the Pacific region spanning southern California to the Baja California Peninsula, the phylogeographic patterns of *Tylos* in the region encompassing the Gulf of California and central Pacific Mexico are deeper and more complex. Multiple highly divergent lineages are observed, indicating long-standing isolation of numerous populations. Deep phylogeographic breaks may be related to vicariant events associated with the formation of the Gulf of California and the Baja California Peninsula. Unfortunately, two issues severely limit our ability to interpret phylogeographic patterns. First, key aspects of the complex geological history of this region remain controversial (reviewed in [6]). Second, obtaining reliable divergence date estimates for the nodes in the phylogeny of *Tylos* is not feasible, because well-established calibration points (e.g. fossils or vicariant events) are not available, and the substitution rates of *Tylos* are unknown.

Two main stages are recognized during the evolution of the Gulf of California [17], [89]. The first stage involves the presence of a northern proto-Gulf basin, which occupied the northern portion of today's Gulf and an extensive area to the north [90], and is estimated to have existed at least 11.61 Ma [16]. A Late Miocene seaway that connected the Pacific with the proto-Gulf basin through the central part of Baja has been proposed [17], [91], [92]. During the second stage, the Gulf of California-Baja Peninsula region attained its present form [89]. The existence of a southern basin 5.5–3.5 Ma is suggested [93], which then joined the northern proto-Gulf to form the present-day Gulf [10]. Accordingly, some extant Gulf lineages may have colonized and remained in the Gulf since northern proto-Gulf times [6]. The Baja Peninsula Cape region is suggested to have been the last portion of the peninsula to separate from mainland, when the modern mouth of the Gulf formed [94], [95]. Some geologists, however, consider that marine incursions in the southern portion of the Gulf of California occurred earlier (∼8 Ma) than in the northern portion (∼6.5 Ma) [96]. They propose that the formation of the Gulf and separation of the Baja Peninsula proceeded from south (i.e., the present-day mouth) to north.

Colonization of the Gulf by *Tylos* was likely achieved by the ancestor of *Clade C–I*; a Gulf-endemic clade. The deep divergences observed within *Clade C–I* (up to 17.3% K2P COI; Table S7) suggest that colonization of the Gulf by *Tylos* may have occurred at early stages of the Gulf formation (possibly as early as proto-Gulf times). For comparative purposes, K2P substitution rates of 2.5%/My for COI and 0.43%/My for 16S rDNA have been reported for an aquatic and a marine isopod, respectively [97], [98].

Colonization of the Gulf by *Tylos*, however, may have been achieved earlier by the ancestor of *Clade B–I*. If so, the sister group to Mazatlan (e.g. Manzanillo to Zihuatanejo), in *Clade B*, may represent a subsequent colonization of the area south of the Gulf. Interestingly, in *Ligia*, a phylogeographic break (>3% K2P COI) is observed between a shallow clade encompassing the localities from Manzanillo to Zihuatanejo, and a clade involving localities further north along the Gulf’s mainland coast, including Mazatlan [6]. The shallow divergences observed in the Manzanillo to Zihuatanejo clades of both *Ligia* and *Tylos* (<1% and ∼1.3% K2P COI; respectively) suggest that lineages from both species recently expanded in this area (∼250 km of coastline).

*Lineage C* is highly divergent (>12.5% K2P COI) and was found only at a small beach ∼14 Km south of Loreto. The limited distribution of this lineage suggests that either it has not dispersed from this restricted area or it has gone extinct in other areas. Past isolation of the Loreto basin may explain the divergence of *Lineage C*. The oldest marine incursions in the Loreto basin are estimated at ∼2.4–2.0 Ma [99] or <3.3 Ma [17]. Another possibility is that this lineage diverged in other basins and subsequently colonized the Loreto area. Marine deposits 6.0–5.3 Ma old are found in Carmen and Monserrat islands in the proximity of Loreto Bay, whereas the Santa Rosalia basin, north of Loreto in the central Peninsula, has an age of ∼10–8 Ma [100].

*Lineage D* is another highly divergent lineage that appears to have a very restricted distribution, as it was only found in Ceuta, north of Mazatlan. The Gulf mainland region between Guaymas and Ceuta, however, has been poorly explored for *Tylos*. Further examination of this region is needed to assess whether *Lineage D* has a broader distribution. We searched several localities along this stretch, but could not find *Tylos*. We note that obtaining the single specimen collected in Ceuta demanded a major searching effort.

The deep divergence between *Clades E* and *F* (>11% K2P COI) suggests a long presence of *Tylos* in the northern Gulf of California. Interestingly, both clades exhibit splits involving a lineage containing Bahia de los Angeles and a lineage containing the midriff island of Salsipuedes (and Angel de la Guarda in the case of *Clade F*). These independent splits share similar divergences (5.8–7.7% K2P COI for *Clade E* and 6.2–6.9% for *Clade F*), which might reflect a common vicariant or dispersal event. Separation of midriff islands in the proximity of Bahia de los Angeles (i.e., Angel de la Guarda, Salsipuedes, and San Lorenzo) could have provided opportunities for such an event. The island of Angel de la Guarda is suggested to have separated from the Puertecitos area (∼190 km NW of Bahia de los Angeles) ∼3–2 Ma and migrated southeast to its current position [101]. The San Lorenzo Archipelago was located across from Bahia Las Animas and Sierra Las Animas, just south of Bahia de los Angeles, during Pliocene time, before its southeastward migration [102]. The basin located between the eastern Sierra Las Animas and the San Lorenzo Archipelago is suggested to have formed during the late Miocene–early Pliocene, ∼8–4 Ma [102]. Dispersal, however, may have occurred between populations of Angel de la Guarda and Salsipuedes, and between these and populations at non-insular localities with which they are closely related. For example, the close relationship of Angel de la Guarda and Salsipuedes to San Lucas appears to be the result of dispersal.

The shallower divergences of *Clade GHI* (<7% K2P COI) compared to *Clade DEF* suggest that diversification within *Clade GHI* is more recent, possibly after proto-Gulf times (i.e., once the present-day peninsula was completely formed). *Clade GHI*, however, has a broader distribution and appears to have a higher number of lineages. The three main clusters within this clade (i.e., *G*, *H*, and *I*) have regional and allopatric distributions. *Clade G*, which is distributed in the upper half of the Gulf, has the highest diversity of lineages and broadest distribution. *Clade I* has a more limited distribution in the central part of the peninsula, in the region between Loreto and Santa Rosalia. This region includes a series of basins that have separated at different times and may have contributed to the isolation and differentiation of *Tylos* lineages. These include the basins of Santa Rosalia, Bahia Concepcion, San Nicolas, and Loreto [95], [100], [103], [104]. Divergences within *Clade I* are <2%, however, suggesting a recent expansion. *Clade H* is restricted to the Cape region of the peninsula. Past vicariant events that occurred around the mid-Peninsula and the Cape region (reviewed in [6]), may explain the divergence of clades *G*, *H*, and *I*.

As observed in the Pacific region spanning southern California to the Baja California Peninsula, the phylogeographic patterns of *Tylos* within the Gulf of California are also different to those of *Ligia* [6]. Within the Gulf of California, *Ligia* shows two reciprocally monophyletic clades that are highly divergent (15–26% COI K2P). One is distributed in the northern Gulf (*Clade Gulf North*) and the other in the southern Gulf and Central Pacific Mexico (*Clade Gulf South*). Lineages in the Gulf’s southern half of the Peninsula are, thus, most closely related to mainland lineages between the central Gulf and central Pacific Mexico. Moreover, lineages from the southernmost portion of the peninsula (Baja Cape Region) are most closely related to the southernmost portion of mainland. Hurtado et al. [6] suggest that the divergence between the two main Gulf clades probably occurred during the Miocene, and that the *Clade Gulf North* represents a lineage that colonized the northern proto-Gulf. They also indicate that a closer relationship between Cape region and southern mainland lineages is congruent with the hypothesis that the Cape region was the last portion of the peninsula to separate from mainland [94], [95]. Such patterns, however, were not observed in *Tylos*. The lineages of *Tylos* found in the southern portion of the peninsula (*Clades H* and *I*) are not more closely related to any lineage in the mainland. Nevertheless, it is possible that such a lineage is present in the southern mainland Gulf portion that was poorly examined (i.e., Guaymas to Ceuta). One similar phylogeographic pattern between *Tylos* and *Ligia*, however, is *Clade EF* of *Tylos* and *Clade Gulf North* of *Ligia*, both of which are relatively deep and approximately co-distributed in the upper half of the Gulf.

Another difference between *Ligia* and *Tylos* is that *Ligia* exhibits almost complete allopatry of divergent lineages [6], whereas two or three divergent lineages of *Tylos* co-occur at multiple localities, particularly in the upper half of the Gulf. This was observed in: Puerto Libertad (*Clades E* and *G*); La Choya (*Clades E* and *G*); San Luis Gonzaga (*Clades E* and *G*); Bahia de los Angeles (*Clades E*, *F*, and *G*); Salsipuedes (*Clades E* and *F*); and Loreto (*Clades C* and *I*). Remarkably, in La Choya, we collected *Clade G* specimens in July 2009, and *Clade E* specimens at exactly the same location in August 2010. Co-occurrence of divergent lineages of *Tylos* was likely achieved by post-divergence dispersal. Dispersal might have been facilitated during low sea level periods, when the northern Gulf basin and coastline were greatly contracted relative to present-day, due to the extensive continental shelf of this region [105]. Two phenomena may explain the higher co-occurrence of lineages of *Tylos* compared to *Ligia*. First, habitat connectivity for *Tylos* is greater than for *Ligia*, because continuous sandy stretches are more widespread than the relatively discrete rocky supralittoral patches in the Gulf of California. Furthermore, rocky patches resting on sandy substrates may not constitute effective dispersal barriers for *Tylos*, which may move across them, as observed during our field surveys. Secondly, competitive exclusion [106] may prevent the successful establishment of newly arrived lineages of *Ligia* at rocky beaches already occupied by a divergent congener. In contrast, it is possible that greater availability of sandy habitat mitigates competitive exclusion among lineages of *Tylos*. In this regard, *Ligia* is relatively abundant and easy to find in rocky habitats, whereas the abundance and density of *Tylos* in sandy beaches appears to be relatively lower.

### 4.3 Conservation Implications

The high levels of morphologically cryptic diversity detected for *Tylos* in this study bring about challenges for the conservation of these isopods in the study area. Local-level efforts will be necessary for the conservation of the multiple divergent lineages with restricted distributions that occur in the Gulf of California and south of this basin. Unfortunately, *Tylos* isopods are very vulnerable to human activities that cause disturbances in sandy beaches [5], which are rapidly increasing in this region, as human populations and tourism expand [31], [32]. These include destruction and modification of sandy beaches, pollution of both terrestrial and marine environments (e.g. from sewage, agriculture, mining, energy production, and transportation), and global change [3]. Furthermore, natural processes such as storms and hurricanes, which are frequent in the Gulf of California, can also have dramatic impacts on the habitat and populations of *Tylos* (Hurtado, personal observation; [3]). In addition, the relatively low reproductive rate of this isopod, compared with other oniscideans, makes this organism even more vulnerable. In the Pacific coast of Baja California, close to Ensenada, females reach reproductive maturity at 3 years, and produce a single brood (4–20 young; average 13.6), after which they usually die [30].

The rapid extirpation of southern California populations of *Tylos* underscores the vulnerability of these isopods to habitat loss and alteration associated with coastal development and beach management practices. In the ∼450 km of shoreline of this region, 16 populations have been eliminated from 28 sites where they were historically reported since the early last century [33]. The northern range limit of *Tylos* in this region shifted south by 31 km since 1971; and abundances of the surviving populations have declined drastically on the mainland coast [33]. Our results suggest this region is occupied by a single species, *T. punctatus*, which is distributed from southern California to the Central Pacific Baja California coast. According to the very low genetic divergence identified within this clade (i.e., *Clade A*), it is very unlikely that the extinct populations in southern California correspond to highly differentiated endemic lineages. Nonetheless, it is possible that some populations within this clade are genetically distinct, as contemporaneous gene flow among *Tylos* populations is likely restricted given the low vagility of this isopod. As mentioned above, this isopod appears to have experienced a recent expansion in this region that was probably facilitated during the period in which the present-day sand-dominated coastlines of the Southern California Bight developed (i.e., 4000–6000 ya [9]). Isolation of populations in this region, thus, appears to be relatively recent. Population genetic studies based on rapidly evolving markers (e.g. microsatellites) are urgently needed to identify potentially distinct populations, assess their genetic health, and facilitate their protection.

Sandy beach communities are largely overlooked for conservation efforts [107]. Protection of *Tylos* isopods can benefit sandy shores and other organisms associated with this habitat, which are in general poorly known [18]. Direct-developing invertebrates, which are expected to have a limited dispersal potential, may account for >50% of all intertidal species found on sandy beaches in California and Washington [108]. *Tylos*, which has these characteristics, can potentially be used as an indicator species regarding the ecosystem health of supralittoral sandy beach communities. In addition, *Tylos* also appears well suited for use as a biomonitor organism of sandy beach contamination from both terrestrial and marine origin. Concentrations of heavy metals and pesticides in *Tylos* collected concurrently with the Gulf of California specimens used in the present study reflected human activities and natural features (Hernández-Garcia and Hurtado, unpublished).

## Supporting Information

Figure S1 **Neighbor-Joining tree of 154 taxa including 1–9 individuals per locality, based on the mitochondrial 16S rDNA and COI gene fragments (Dataset S1).** Red taxon names indicate the subset of samples examined for the phylogenetic analyses of four mitochondrial and two nuclear gene fragments (Dataset S2).
(PDF)

Figure S2 **Majority-rule consensus tree (RaxML bootstrap) of the study area dataset based on concatenated mitochondrial loci (MT).** Colors and shapes correspond to clades in other figures. Numbers by nodes indicate the corresponding range of Bootstrap Support (BS; top or left) for Maximum likelihood (RaxML, Garli, PartitionFinder); and Posterior Probabilities (PP; bottom or right) for Bayesian inference methods (MrBayes, Phycas, BayesPhylogeny), including all partitioning schemes. * denotes nodes that received 100% support for all methods. Nodes receiving less than 50% support for all methods were collapsed and denoted with <50. † = relationship based on 16S sequence only: Isla Angel de la Guarda.
(PDF)

Figure S3 **Majority-rule consensus trees (RaxML bootstrap) of the study area dataset based on 18S rDNA gene.** Colors and shapes correspond to clades in other figures. Numbers by nodes indicate the corresponding range of Bootstrap Support (BS; top or left) for Maximum likelihood (RaxML, Garli, PartitionFinder); and Posterior Probabilities (PP; bottom or right) for Bayesian inference methods (MrBayes, Phycas, BayesPhylogeny), including all partitioning schemes. * denotes nodes that received 100% support for all methods. Nodes receiving less than 50% support for all methods were collapsed and denoted with <50.
(PDF)

Figure S4 **Majority-rule consensus trees (RaxML bootstrap) of the study area dataset based on Histone gene (H3A).** Colors and shapes correspond to clades in other figures. Numbers by nodes indicate the corresponding range of Bootstrap Support (BS; top or left) for Maximum likelihood (RaxML, Garli, PartitionFinder); and Posterior Probabilities (PP; bottom or right) for Bayesian inference methods (MrBayes, Phycas, BayesPhylogeny), including all partitioning schemes. * denotes nodes that received 100% support for all methods. Nodes receiving less than 50% support for all methods were collapsed and denoted with <50.
(PDF)

Figure S5 **Photographs of the ventral shape of the fifth pleonite for:** ***Tylos punctatus*** **syntype;** ***Tylos*** **specimens from the study area representing clades** ***A, B, C, E, F, G, H, I*** **; and** ***Tylos niveus*** **(outgroup).** A drawing of this structure in *Tylos insularis* is also shown.
(PDF)

Table S1 (DOCX)

Table S2 (DOCX)

Table S3 (DOCX)

Table S4 (DOCX)

Table S5 (DOCX)

Table S6 (DOCX)

Table S7 (DOCX)

Dataset S1 (NEX)

Dataset S2 (NEX)
