# Supplementary figures and images for: Contrasting Phylogeography of Sandy vs. Rocky Supralittoral Isopods in the Megadiverse and Geologically Dynamic Gulf of California and Adjacent Areas
Source: PLoS One. 2013 Jul 2;8(7):e67827. doi: 10.1371/journal.pone.0067827 (PMC3699670; doi:10.1371/journal.pone.0067827)

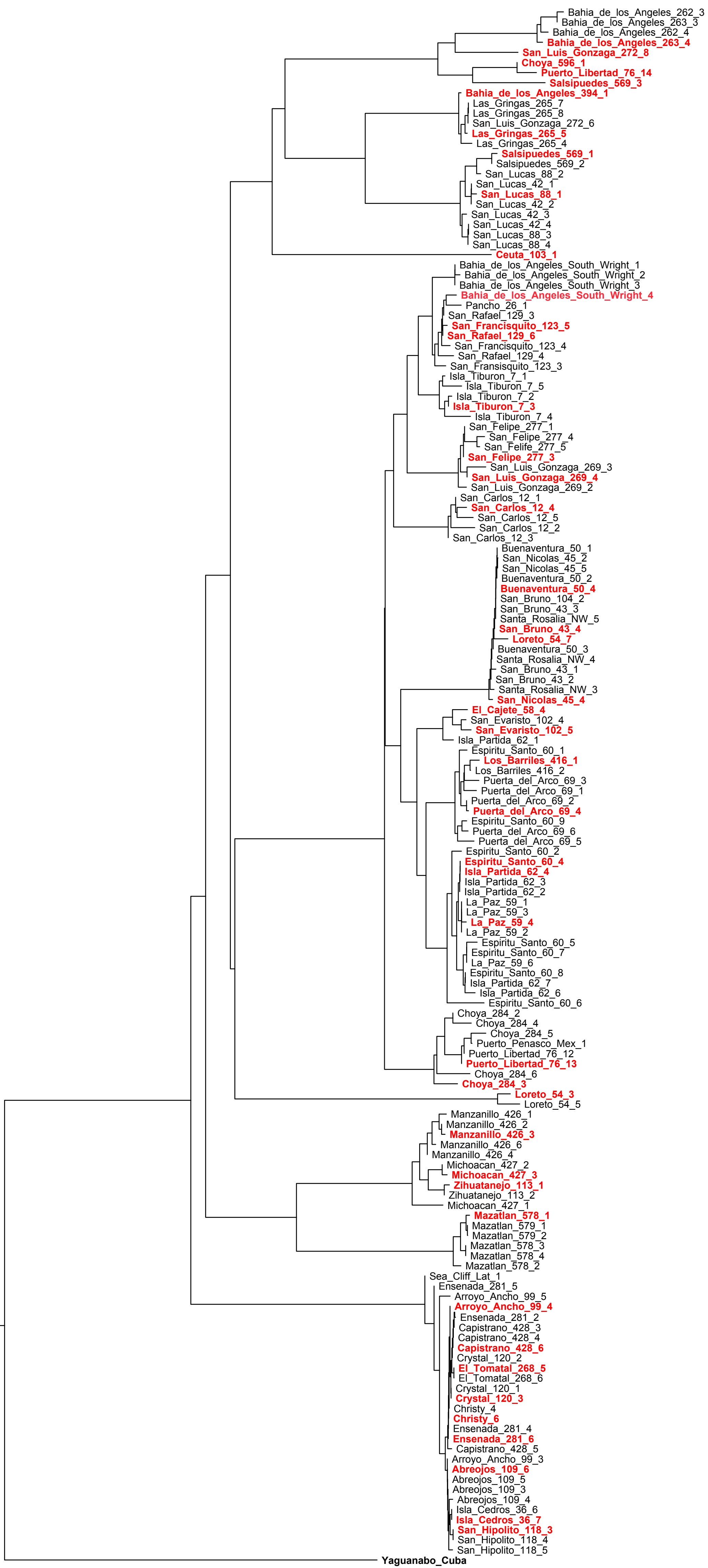

0.02

Supplement: Figure S1 — Neighbor-Joining tree of 154 taxa including 1–9 individuals per locality, based on the mitochondrial 16S rDNA and COI gene fragments (Dataset S1). Red taxon names indicate the subset of samples examined for the phylogenetic analyses of four mitochondrial and two nuclear gene fragments (Dataset S2). (PDF) [file pone.0067827.s001.pdf]

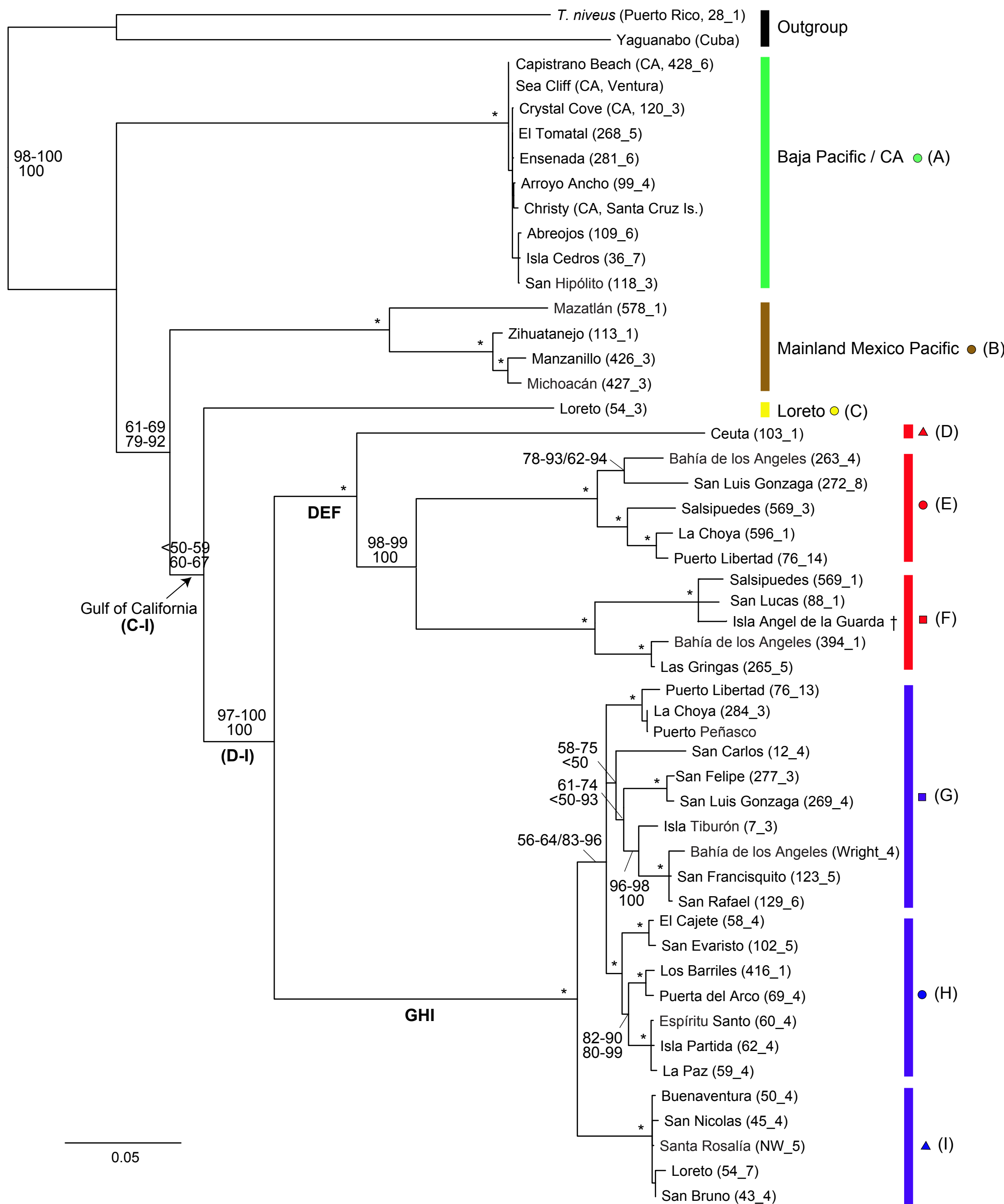

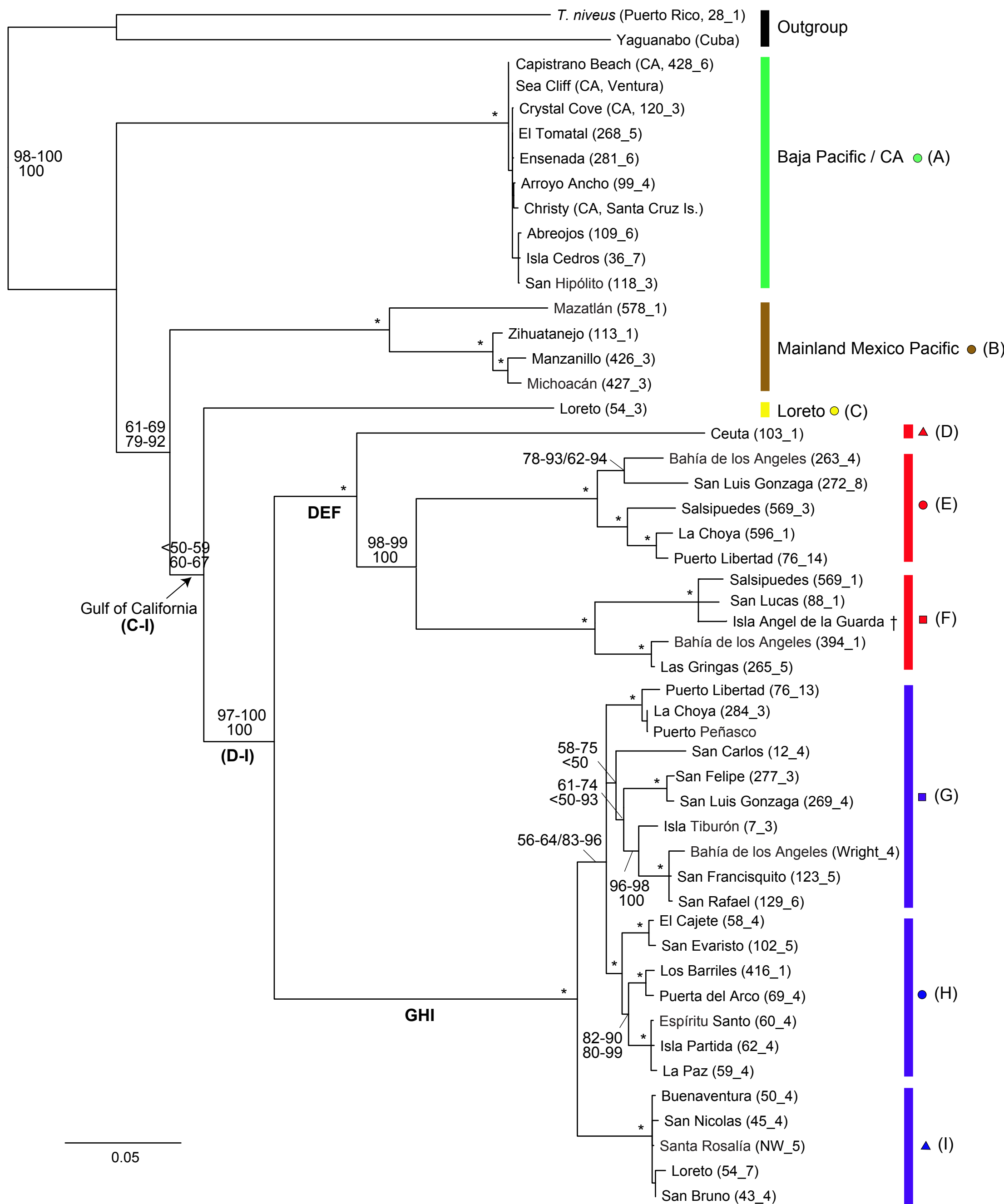

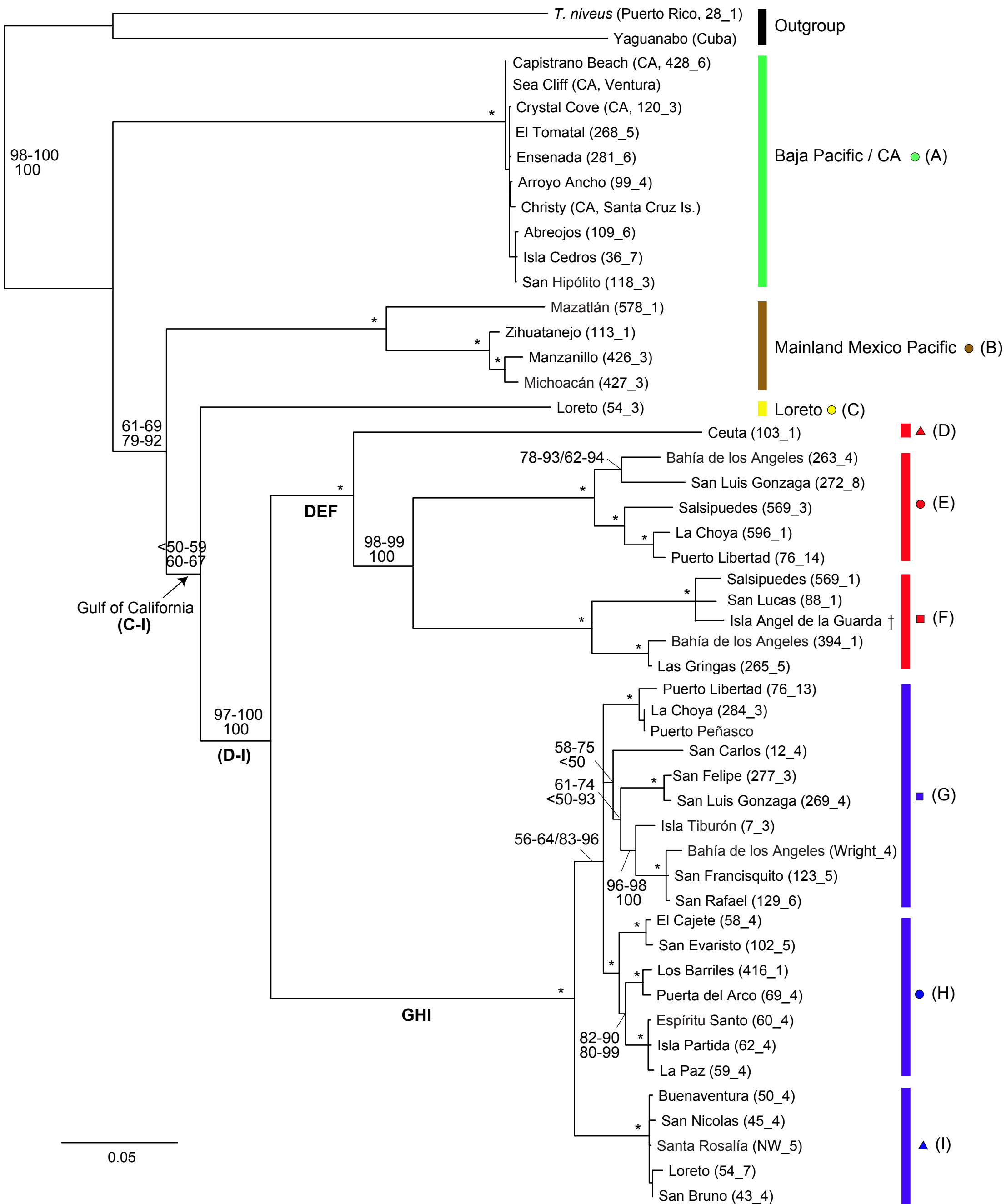

Supplement: Figure S2 — Majority-rule consensus tree (RaxML bootstrap) of the study area dataset based on concatenated mitochondrial loci (MT). Colors and shapes correspond to clades in other figures. Numbers by nodes indicate the corresponding range of Bootstrap Support (BS; top or left) for Maximum likelihood (RaxML, Garli, PartitionFinder); and Posterior Probabilities (PP; bottom or right) for Bayesian inference methods (MrBayes, Phycas, BayesPhylogeny), including all partitioning schemes. * denotes nodes that received 100% support for all methods. Nodes receiving less than 50% support for all methods were collapsed and denoted with <50. † = relationship based on 16S sequence only: Isla Angel de la Guarda. (PDF) [file pone.0067827.s002.pdf]

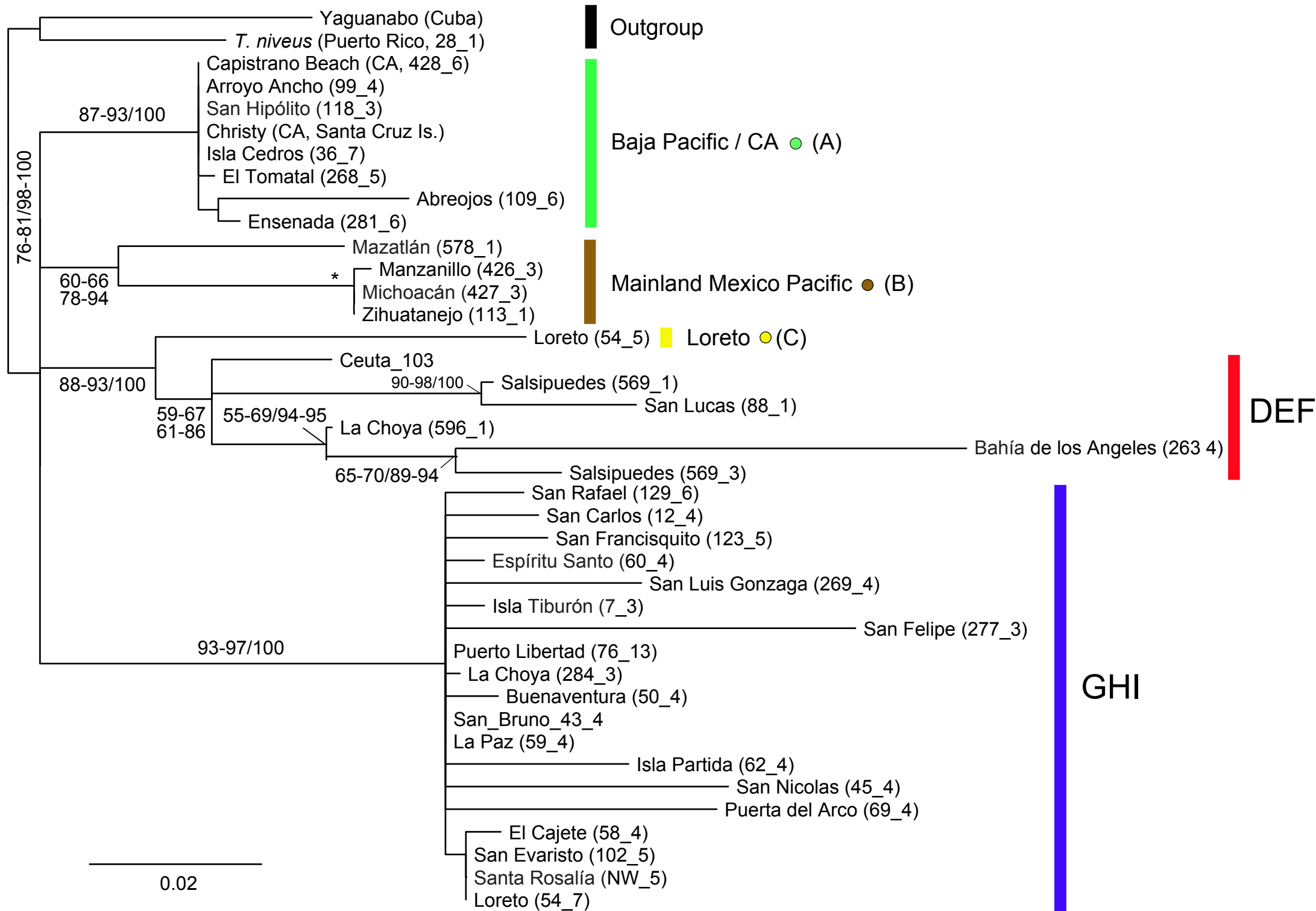

Supplement: Figure S3 — Majority-rule consensus trees (RaxML bootstrap) of the study area dataset based on 18S rDNA gene. Colors and shapes correspond to clades in other figures. Numbers by nodes indicate the corresponding range of Bootstrap Support (BS; top or left) for Maximum likelihood (RaxML, Garli, PartitionFinder); and Posterior Probabilities (PP; bottom or right) for Bayesian inference methods (MrBayes, Phycas, BayesPhylogeny), including all partitioning schemes. * denotes nodes that received 100% support for all methods. Nodes receiving less than 50% support for all methods were collapsed and denoted with <50. (PDF) [file pone.0067827.s003.pdf]

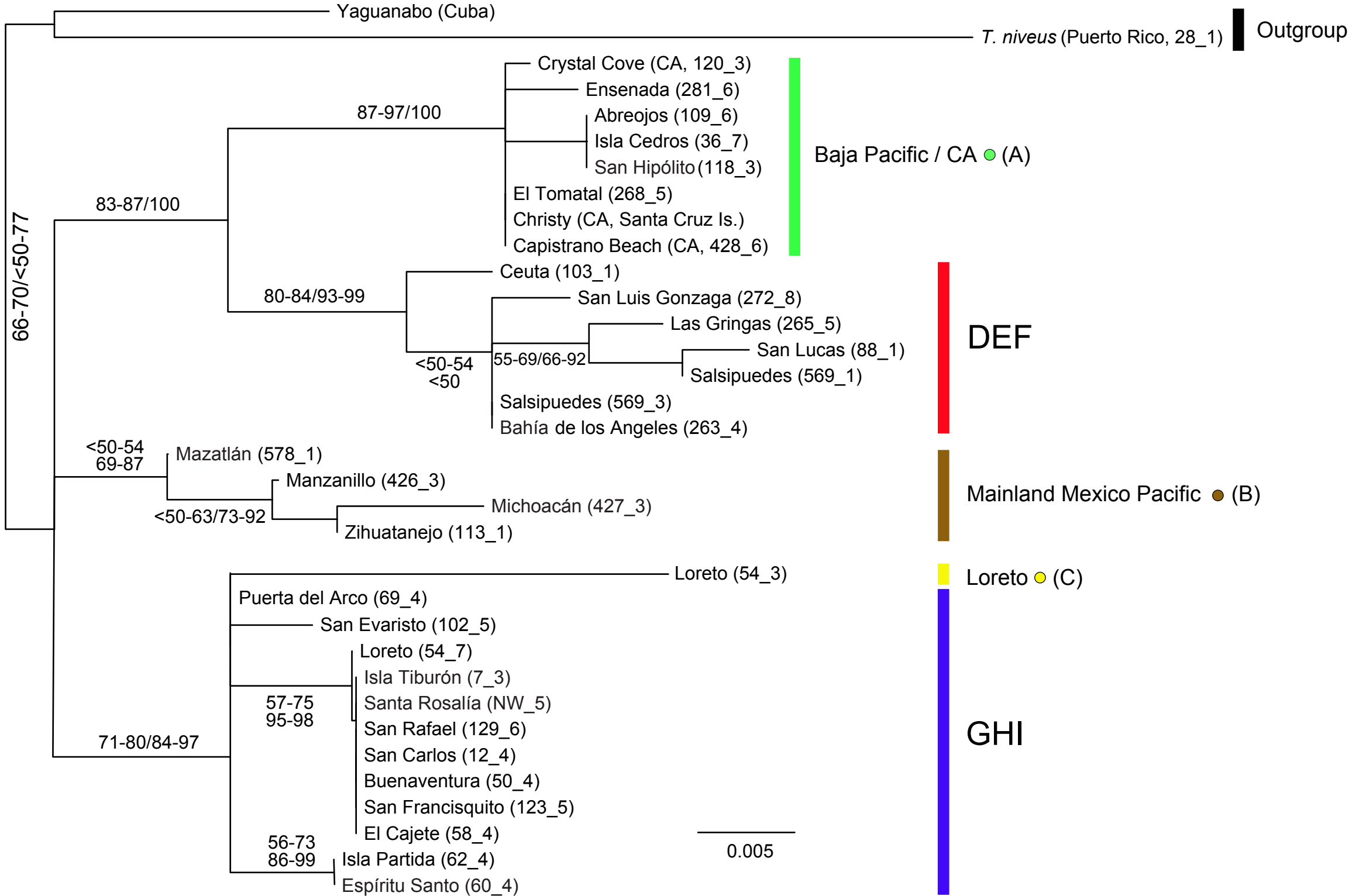

Supplement: Figure S4 — Majority-rule consensus trees (RaxML bootstrap) of the study area dataset based on Histone gene (H3A). Colors and shapes correspond to clades in other figures. Numbers by nodes indicate the corresponding range of Bootstrap Support (BS; top or left) for Maximum likelihood (RaxML, Garli, PartitionFinder); and Posterior Probabilities (PP; bottom or right) for Bayesian inference methods (MrBayes, Phycas, BayesPhylogeny), including all partitioning schemes. * denotes nodes that received 100% support for all methods. Nodes receiving less than 50% support for all methods were collapsed and denoted with <50. (PDF) [file pone.0067827.s004.pdf]
