# Supplementary material for: Contrasting Phylogeography of Sandy vs. Rocky Supralittoral Isopods in the Megadiverse and Geologically Dynamic Gulf of California and Adjacent Areas
Source: PLoS One. 2013 Jul 2;8(7):e67827. doi: 10.1371/journal.pone.0067827 (PMC3699670; doi:10.1371/journal.pone.0067827)
Supplement: Figure S5 — Photographs of the ventral shape of the fifth pleonite for: Tylos punctatus syntype; Tylos specimens from the study area representing clades A, B, C, E, F, G, H, I ; and Tylos niveus (outgroup). A drawing of this structure in Tylos insularis is also shown. (PDF) [file pone.0067827.s005.pdf]

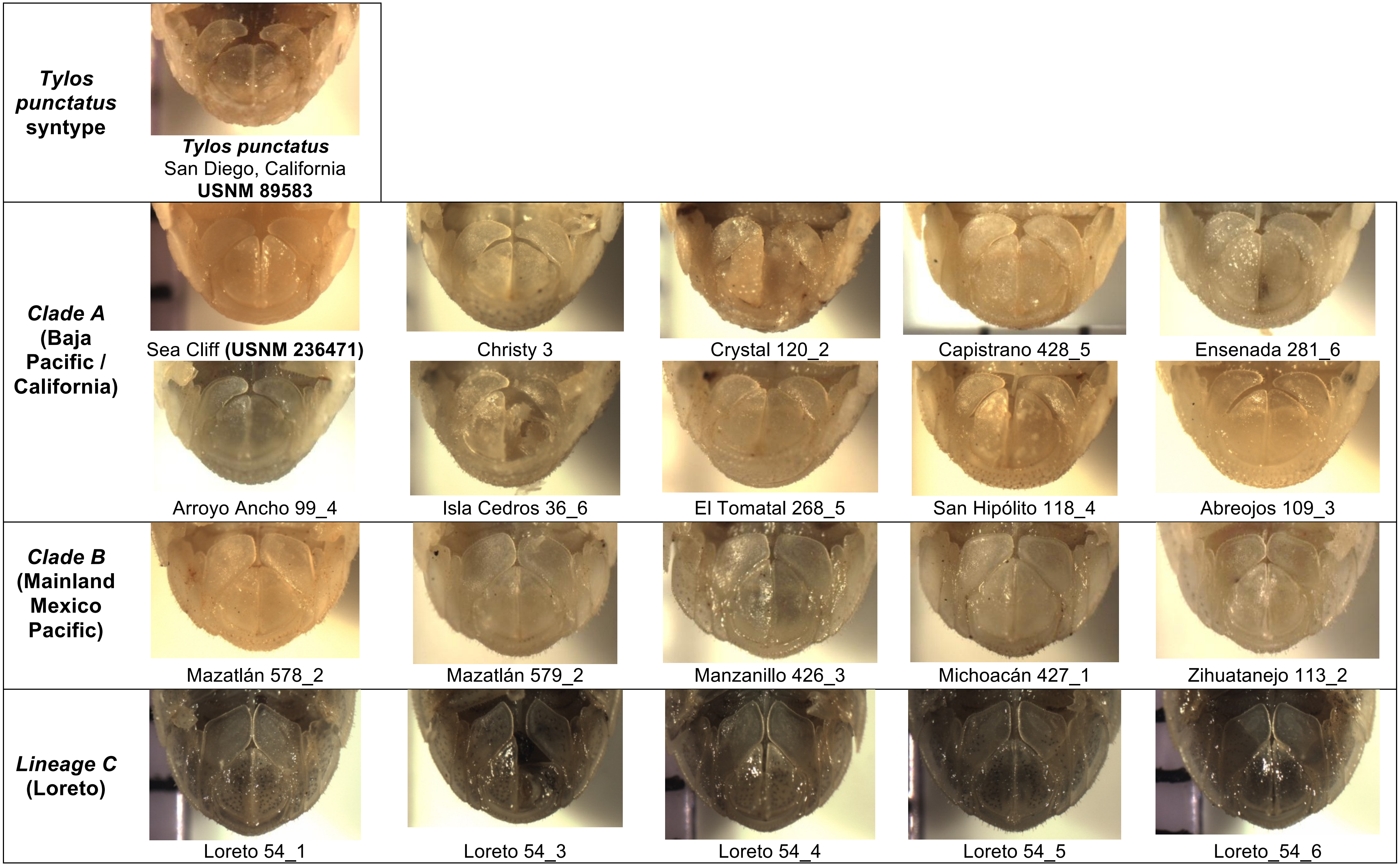

Tylos punctatus syntype
Tylos punctatus
San Diego, California
USNM 89583
Clade A (Baja Pacific / California)
Sea Cliff (USNM 236471)
Christy 3
Crystal 120_2
Capistrano 428_5
Ensenada 281_6
Arroyo Ancho 99_4
Isla Cedros 36_6
El Tomatal 268_5
San Hipólito 118_4
Abreojos 109_3
Clade B (Mainland Mexico Pacific)
Mazatlán 578_2
Mazatlán 579_2
Manzanillo 426_3
Michoacán 427_1
Zihuatanejo 113_2
Lineage C (Loreto)
Loreto 54_1
Loreto 54_3
Loreto 54_4
Loreto 54_5
Loreto_54_6

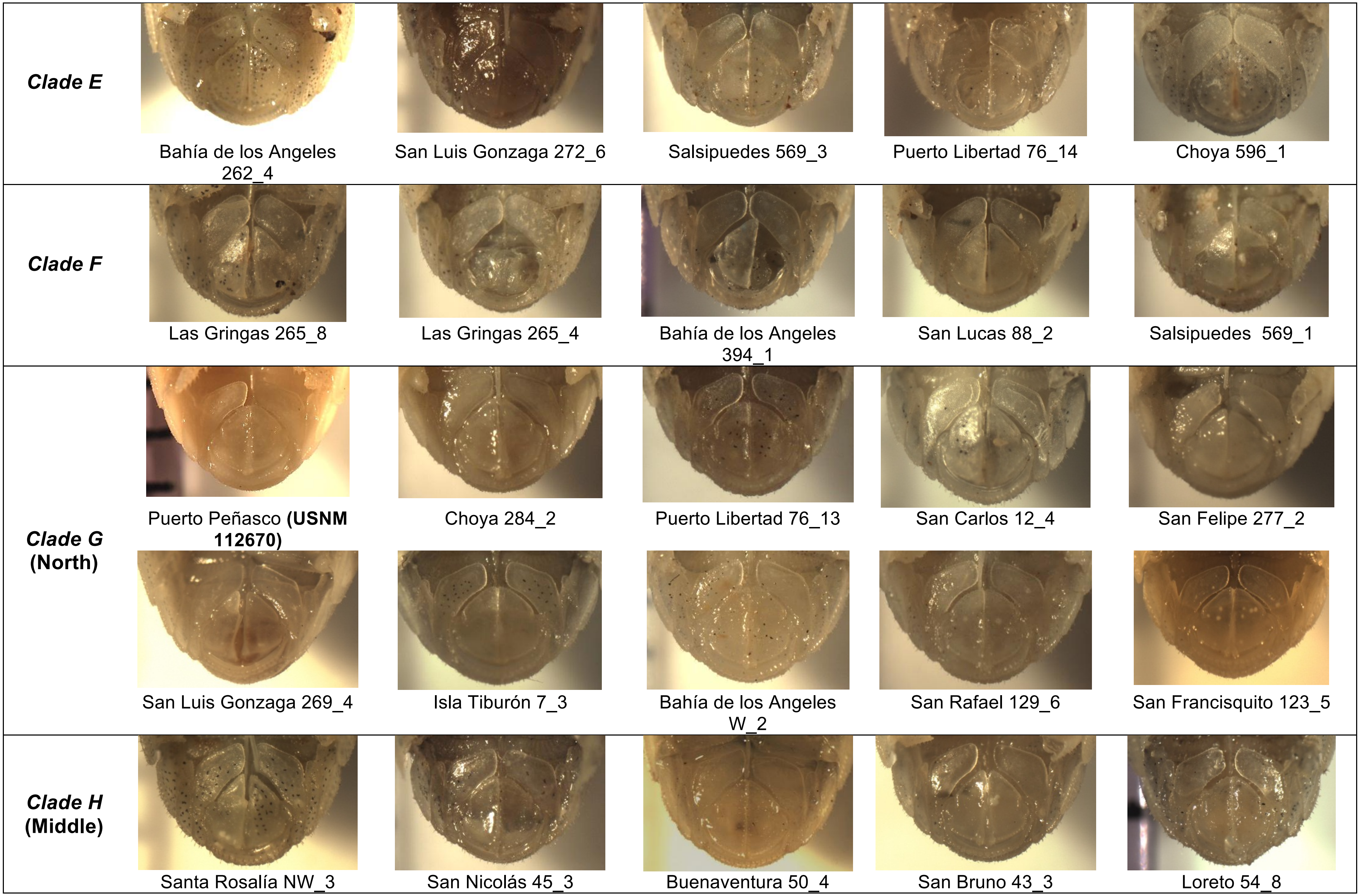

Clade E
Bahía de los Angeles 262_4
San Luis Gonzaga 272_6
Salsipuedes 569_3
Puerto Libertad 76_14
Choya 596_1
Clade F
Las Gringas 265_8
Las Gringas 265_4
Bahía de los Angeles 394_1
San Lucas 88_2
Salsipuedes 569_1
Clade G (North)
Puerto Peñasco (USNM 112670)
Choya 284_2
Puerto Libertad 76_13
San Carlos 12_4
San Felipe 277_2
San Luis Gonzaga 269_4
Isla Tiburón 7_3
Bahía de los Angeles W_2
San Rafael 129_6
San Francisquito 123_5
Clade H (Middle)
Santa Rosalía NW_3
San Nicolás 45_3
Buenaventura 50_4
San Bruno 43_3
Loreto 54_8

***Clade I* (South)**

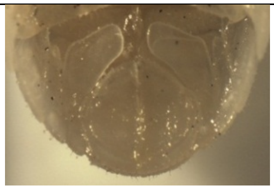

Espíritu Santo 60_3

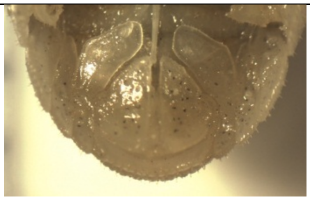

San Evaristo 102_4

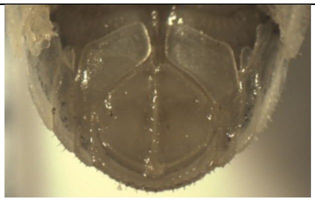

El Cajete 58_4

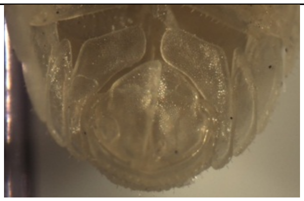

Los Barriles 416_1

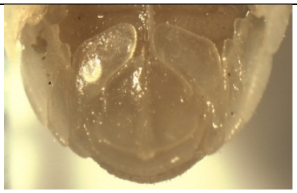

Puerta del Arco 69_4

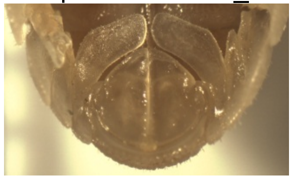

Puerta del Arco 69_2

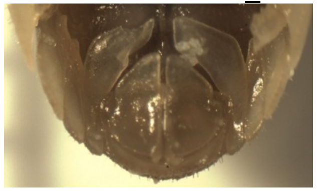

La Paz 59_4

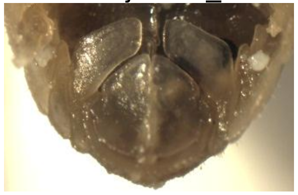

La Paz 59_1

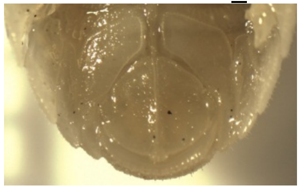

Isla Partida 62_4

Other

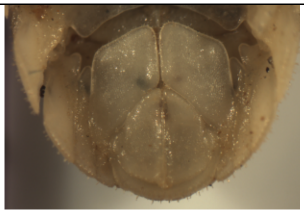

Tylos niveus
28_1, Puerto Rico
(Aguada)

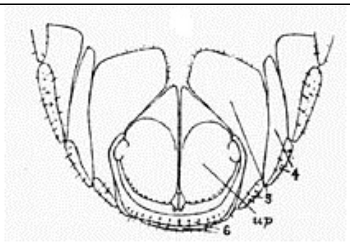

Tylos insularis
(Galapagos)[1]

References

1. Van Name WG (1924) Isopods from the Williams Galapagos Expedition. Zoologica 5: 181–210
