# Supplementary material for: Contrasting Phylogeography of Sandy vs. Rocky Supralittoral Isopods in the Megadiverse and Geologically Dynamic Gulf of California and Adjacent Areas
Source: PLoS One. 2013 Jul 2;8(7):e67827. doi: 10.1371/journal.pone.0067827 (PMC3699670; doi:10.1371/journal.pone.0067827)
Supplement: Table S1 — (DOCX) [file pone.0067827.s006.docx]

**Table S1**

Information on *Tylos* localities examined in the present study (i.e., between Central Mexico to Southern California, including the Gulf of

California). The COI and 16S columns correspond to the number of individuals sequenced for each of these genes.

| **NO** | **COI** | **16S** | **Localities (ID No.)** | **State** | **Country** | **Latitude** | **Longitude** | **Date** |
| --- | --- | --- | --- | --- | --- | --- | --- | --- |
| 1 | 4 | 4 | Abreojos (109) | Baja California Sur | Mexico | 26°44'1.3N | 113°32'58.1W | 07/18/2009 |
| 2 | 3 | 3 | Arroyo Ancho (99) | Baja California | Mexico | 29°54'44.88N | 115°42'40.50W | 08/19/2009 |
| 3 | 1 | 1 | Bahía de los Angeles (394) | Baja California | Mexico | 28°56'19.7N | 113° 33'7.3W | 08/16/2009 |
| 4 | 4 | 4 | Bahía de los Angeles South (Wright) | Baja California | Mexico | 28°50'37.25N | 113°28'23.37W | 06/19/2009 |
| 5 | 4 | 4 | Bahía de los Angeles (263/262) | Baja California | Mexico | 28°54'19.62N | 113°32'2.40W | 07/19/2009 |
| 6 | 4 | 4 | Buenaventura (50) | Baja California Sur | Mexico | 26°38'38.88N | 111°50'46.68W | 11/19/2008 |
| 7 | 4 | 5 | Capistrano Beach (428) | California | USA | 33°29'19.4N | 117°40'13.6W | 08/28/2009 |
| 8 | 1 | 1 | Ceuta (103) | Sinaloa | Mexico | 23°53'39.5N | 106°56'52.2W | 08/25/2009 |
| 9 | 5 | 6 | La Choya (284) | Baja California | Mexico | 31°21'42.8N | 113°38'23.5W | 07/22/2009 |
| 10 | 1 | 1 | La Choya (596) | Baja California | Mexico | 31°21'42.8N | 113°38'23.5W | 08/27/2010 |
| 11 | 2 | 2 | Christy: Santa Cruz (CHR) | California | USA | 34°2'36.8N | 119°51'34.1W | 03/20/2008 |
| 12 | 5 | 4 | Ensenada (281) | Baja California | Mexico | 31°47'23.40N | 116°36'57.36W | 07/22/2009 |
| 13 | 3 | 3 | Crystal Cove Beach (120) | California | USA | 33°34'15.4N | 117°50'14.9W | 06/07/2010 |
| 14 | 6 | 3 | El Cajete (58) | Baja California Sur | Mexico | 24°15'21.06N | 110°36'41.82W | 11/21/2008 |
| 15 | 2 | 2 | El Tomatal (268) | Baja California Sur | Mexico | 28°29'12.5N | 114°4'5W | 11/10/2009 |
| 16 | 2 | 2 | Isla Cedros (36) | Baja California | Mexico | 28°7'8.22N | 115°20'34.80W | 11/14/2008 |
| 17 | 9 | 9 | Isla Espíritu Santo (60) | Baja California Sur | Mexico | 24°24'13.08N | 110°20'53.28W | 11/23/2008 |
| 18 | 7 | 8 | Isla Partida (62/63) | Baja California Sur | Mexico | 24°31'56.58N | 110°23'0.00W | 11/23/2008 |
| 19 | 5 | 5 | Isla Tiburón (7) | Sonora | Mexico | 28°52'57.42N | 112°34'3.90W | 10/29/2008 |
| 20 | 6 | 5 | La Paz (59) | Baja California Sur | Mexico | 24°13'45.60N | 110°18'33.54W | 11/22/2008 |
| 21 | 4 | 4 | Las Gringas (265) | Baja California | Mexico | 29°1'20.82N | 113°33'38.94W | 07/20/2009 |
| 22 | 5 | 4 | Loreto (54) | Baja California Sur | Mexico | 25°52'43.50N | 111°20'31.56W | 11/20/2008 |
| 23 | 2 | 2 | Los Barriles (416) | Baja California Sur | Mexico | 23°44'4.14N | 109°42'46.92W | 11/26/2008 |
| 24 | 6 | 6 | Mazatlán (578/579) | Sinaloa | Mexico | 23°13'54.8N | 106°24'26.6W | 08/28/2009 |
| 25 | 3 | 3 | Michoacán (427) | Michoacán | Mexico | 18°34'31.9N | 103°39'53.1W | 08/28/2009 |
| 26 | 5 | 5 | Manzanillo (426) | Colima | Mexico | 19°6'19.68N | 104°21'6.24W | 08/28/2009 |
| 27 | 6 | 6 | Puerta del Arco (69) | Baja California Sur | Mexico | 23°53'43.7N | 109°48'9.9W | 11/29/2008 |
| 28 | 5 | 3 | Puerto Libertad (76) | Sonora | Mexico | 29°54'9.61N | 112°43'36.31W | 12/02/2008 |
| 29 | 6 | 3 | Salsipuedes (569) | Baja California | Mexico | 28°43'29.7N | 112°57'8.9W | 08/19/2010 |
| 30 | 5 | 6 | San Bruno (43/104/405) | Baja California Sur | Mexico | 27°9'46.26N | 112°9'34.14W | 11/16/2008 |
| 31 | 6 | 5 | San Carlos (12) | Sonora | Mexico | 27°56'21.42N | 111°5'19.26W | 11/02/2008 |
| 32 | 4 | 3 | San Evaristo (102) | Baja California Sur | Mexico | 24°21'59.8N | 110°40'48.7W | 08/22/2009 |
| 33 | 5 | 5 | San Felipe (277) | Baja California | Mexico | 31°1'31.97N | 114°49'52.01W | 07/21/2009 |
| 34 | 3 | 3 | San Hipólito (118) | Baja California Sur | Mexico | 26°59'26.76N | 113°58'38.94W | 07/18/2009 |
| 35 | 8 | 8 | San Lucas (88/42) | Baja California Sur | Mexico | 27°13'31.6N | 112°12'32.9W | 07/17/2009 |
| 36 | 4 | 3 | San Luis Gonzaga (269) | Baja California | Mexico | 29°47'43.32N | 114°23'47.16W | 07/20/2009 |
| 37 | 4 | 2 | San Luis Gonzaga (272) | Baja California Sur | Mexico | 29°47'43.32N | 114°23'47.16W | 07/20/2009 |
| 38 | 4 | 4 | San Nicolás (45) | Baja California Sur | Mexico | 26°32'37.02N | 111°32'24.08W | 11/17/2008 |
| 39 | 5 | 5 | San Rafael / Pancho (129/26) | Baja California | Mexico | 28°34'18.78N | 113°7'12.66W | 07/19/2009 |
| 40 | 3 | 4 | San Francisquito (123/125) | Baja California | Mexico | 28°24'25.3N | 112°51'31.8W | 07/19/2009 |
| 41 | 3 | 3 | Santa Rosalía (NW) | Baja California Sur | Mexico | 27°21'40.62N | 112°16'47.82W | 11/16/2008 |
| 42 | 2 | 2 | Zihuatanejo (113) | Guerrero | Mexico | 17°37'29.22N | 101°32'44.40W | 08/29/2009 |
| 43^1^ | 1 | 1 | Sea Cliff (Lat) | California | USA |  |  | 06/00/1935 |
| 44^2^ | 1 | 1 | Puerto Peñasco (Mex) | Sonora | Mexico |  |  | 04/17/1965 |
| 45^3^ | - | 1 | Isla Angel de la Guarda (#06) | Baja California | Mexico |  |  |  |

^1^ Museum specimen: Smithsonian Institution National Museum of Natural History (specimen # USNM 236471)

^2^ Museum specimen: Smithsonian Institution National Museum of Natural History (specimen # USNM 112670)

^3^ Museum specimen: Natural History Museum of Los Angeles County (specimen # LACM 71-540.2)
