# Supplementary material for: Contrasting Phylogeography of Sandy vs. Rocky Supralittoral Isopods in the Megadiverse and Geologically Dynamic Gulf of California and Adjacent Areas
Source: PLoS One. 2013 Jul 2;8(7):e67827. doi: 10.1371/journal.pone.0067827 (PMC3699670; doi:10.1371/journal.pone.0067827)
Supplement: Table S2 — (DOCX) [file pone.0067827.s007.docx]

**Table S2**

PCR primers information and annealing temperature (Tm).

| Gene | Name | Primer sequences | Tm | References |
| --- | --- | --- | --- | --- |
| **16S** | 16s SAR | 5’-CGCCTGTTTATCAAAAACAT-3’ | 48-49 | [[1](#_ENREF_1)] |
|  | 16s SBR | 5’-CCGGTCTGAACTCAGATCACGT-3’ |  |  |
|  | 16S Tyhe-F | 5'-ATATTGACTGTGCTAAGGTAGC-3' |  | [[2](#_ENREF_2)] |
|  | 16S Tyhe-R | 5'-CTTAATCCAACATCGAGGTC-3' |  |  |
|  | 16s TA | 5’-CTG TGC TAA GGT AGC RTA AT-3’ |  | newly designed |
|  | 16s TB | 5’-TTA ARR GTC GAA CAG AC-3’ |  |  |
| **12S** | 12SCRF | 5’-GAGAGTGACGGGCGATATGT-3’ | 48-49 | [[3](#_ENREF_3)] |
|  | 12SCRR | 5’-AAACCAGGATTAGATACCCTATTAT-3’ |  |  |
|  | Crust-12f | 5’-CAGCAKYCGCGGTTAKAC-3’ |  | [[4](#_ENREF_4)] |
|  | Crust-12r | 5’-ACACCTACTWTGTTACGACTTATCTC-3’ |  |  |
| **Cytb** | Cytb151F | 5’-TGTGGRGCNACYGTWATYACTAA-3’ | 48-49 | [[5](#_ENREF_5)] |
|  | Cytb144F | 5’-TGAGSNCARATGTCNTWYTG-3’ |  |  |
|  | Cytb270R | 5’-AANAGGAARTAYCAYTCNGGYTG-3’ |  |  |
|  | Cytb272R | 5’-GCRAANAGRAARTACCAYTC-3’ |  |  |
| **16S2 - ND4** | N4 | 5’-GGAGCTTCAACATGAGCTTT-3’ | 50 | [[6](#_ENREF_6)] |
|  | 16S2 | 5’-GCGACCTCGATGTTGGATTAA-3’ |  |  |
|  | N4-specific2 | 5’-CYCCRWCYTGGAGKCWYTCSGGY-3’ |  | newly designed |
| **COI** | HCO-2198 | 5’-TAAACTTCAGGGTGACCAAAAAATCA-3’ | 50-55 | [[7](#_ENREF_7)] |
|  | LCO-1490 | 5’-GGTCAACAAATCATAAAGATATTGGGG-3’ |  |  |
|  | M13FLCO-1490 | 5’-TGTAAAACGACGGCCAGTGGTCAACAAAT CATAAAGATATTGG-3’ |  |  |
|  | M13RHCO-2198 | 5’-CAGGAAACAGCTATGACTAAACTTCAGGG TGACCAAAAAATCA-3’ |  |  |
|  | HCO-A | 5’-ATT TTG TKT TTG GNG CNT GAG C-3’ |  | newly designed |
|  | LCO-A | 5’-GRT SNC CNC CNC CYC CYC TRG GRT C-3’ |  | newly designed |
| **18S** | 18s-3F | 5’-GTTCGATTCCGGAGAGGGA-3’ | 49 | [[8](#_ENREF_8)] |
|  | 18s-5R | 5’-CTTGGCAAATGCTTTCGC-3’ |  |  |
| **H3** | H3-aF | 5’-ATGGCTCGTACCAAGCAGACVGC-3’ | 49 | [[9](#_ENREF_9)] |
|  | H3-aR | 5'-ATATCCTTRGGCATRATGTGAC-3' |  |  |

**References**

1. Palumbi S (1996) Nucleic Acids II: Polymerase Chain Reaction. In: Hillis D, Moritz C, Mable B, editors. Molecular Systematics. 2nd ed. Sunderland, Massachusetts: Sinauer Associates, Inc. pp. 205-247.

2. Gentile G, Campanaro A, Carosi M, Sbordoni V, Argano R (2010) Phylogeography of *Helleria brevicornis* Ebner 1868 (Crustacea, Oniscidea): old and recent differentiations of an ancient lineage. Mol Phylogenet Evol 54: 640-646

3. Wetzer R (2001) Hierarchical analysis of mtDNA variation and the use of mtDNA for isopod (Crustacea: Peracarida: Isopoda) systematics. Contrib Zool 70: 23-40

4. Podsiadlowski L, Bartolomaeus T (2005) Organization of the mitochondrial genome of mantis shrimp *Pseudosquilla ciliata* (Crustacea: Stomatopoda). Mar Biotechnol 7: 618-624. doi: 10.1007/s10126-005-0017-8

5. Merrit TJS, Shi L, Chase MC, Rex MA, Etter RJ, et al. (1998) Universal cytochrome b primers facilitate intraspecific studies in molluscan taxa. Mol Mar Biol Biotechnol 7: 7-11

6. Roehrdanz RL, Degrugillier ME, Black WC (2002) Novel rearrangements of arthropod mitochondrial DNA detected with long-PCR: applications to arthropod phylogeny and evolution. Mol Biol Evol 19: 841-849

7. Folmer O, Black MB, Hoeh WR, Lutz RA, Vrijenhoek RC (1994) DNA primers for amplification of mitochondrial cytochrome C oxidase subunit I from metazoan invertebrates. Mol Mar Biol Biotechnol 3: 294-299

8. Giribet G, Carranza S, Bagui J, Riutort M, Ribera C (1996) First molecular evidence for the existence of a tardigrada + arthropodaclade. Mol Biol Evol 13: 76-84

9. Colgan DJ, McLauchlan A, Wilson GDF, Livingston SP, Edgecombe GD, et al. (1998) Histone H3 and U2 snRNA DNA sequences and arthropod molecular evolution. Aust J Zool 46: 419-437
