# Supplementary material for: Contrasting Phylogeography of Sandy vs. Rocky Supralittoral Isopods in the Megadiverse and Geologically Dynamic Gulf of California and Adjacent Areas
Source: PLoS One. 2013 Jul 2;8(7):e67827. doi: 10.1371/journal.pone.0067827 (PMC3699670; doi:10.1371/journal.pone.0067827)
Supplement: Table S3 — (DOCX) [file pone.0067827.s008.docx]

**Table S3**

Description of characters and substitution models. Number of characters per gene region that were excluded from and included in the phylogenetic analyses. The number of parsimony informative characters is based on included characters only. Best model selected by jModelTest according to each criterion (AIC, AICc, BIC) and its corresponding weight.

| Gene region | Samples | Total characters^a^ | Excluded characters^b^ | Included characters | Parsimony-informative | AICc (weight) | AIC  (weight) | BIC  (weight) |  |
| --- | --- | --- | --- | --- | --- | --- | --- | --- | --- |
| 16S | 137 | 491 | 145 | 346 | 99 | HKY+I+G (1.00) | GTR+I+G (1.00) | GTR+I+G (1.00) | |
| 12S | 44 | 350 | 74 | 276 | 63 | TIM2+G  (0.29) | TIM2+I+G (0.24) | TrN+G  (0.36) | |
| COI | 113 | 657 | 57 | 600 | 194 | TIM1+I+G (0.95) | TIM1+I+G (0.88) | TIM1+I+G (0.93) | |
| Cytb | 47 | 296 | 0 | 296 | 117 | TIM2+I+G (0.61) | TIM2+I+G (0.63) | TIM2+I+G (0.57) | |
| ND6/4 | 43 | 1639 | 165 | 1474 | 621 | TIM2+I+G (0.91) | TIM2+I+G (0.88) | TIM2+I+G (0.99) | |
| MT | 50**^c^** | 3433 | 441 | 2992 | 1058 | TIM2+I+G (0.66) | TIM2+I+G (0.64) | TrN+I+G (0.49) | |
| H3A | 34 | 285 | 0 | 285 | 20 | TPM1+G (0.25) | TPM1uf+G (0.10) | TPM1+G (0.30) | |
| 18S | 40 | 1121 | 601 | 520 | 110 | TrNef+I+G (0.30) | TrNef+I+G (0.23) | K80+I+G (0.48) | |
| MT+NC | 50**^d^** | 4839 | 1042 | 3797 | 1188 | TIM2+I+G (0.54) | TIM2+I+G (0.54) | HKY+I+G (0.78) | |

**^a^** Total number of characters in the alignment, including gaps.

**^b^** Criteria for character exclusion are described in a nexus file in the supporting information.
**^c^** Includes taxa that were missing one or two mitochondrial genes.

**^d^** Includes taxa that were missing one or more mitochondrial or nuclear genes.

MT = concatenated mitochondrial genes.
