# Supplementary material for: Contrasting Phylogeography of Sandy vs. Rocky Supralittoral Isopods in the Megadiverse and Geologically Dynamic Gulf of California and Adjacent Areas
Source: PLoS One. 2013 Jul 2;8(7):e67827. doi: 10.1371/journal.pone.0067827 (PMC3699670; doi:10.1371/journal.pone.0067827)
Supplement: Table S4 — (DOCX) [file pone.0067827.s009.docx]

**Table S4**

Models, parameters, and priors used in the Maximum Likelihood and Bayesian phylogenetic analyses of the concatenated mitochondrial (MT) and nuclear (NC) dataset.

| Method | Model and Priors^1^ | Partitioning scheme^2^ | iterations generations/bootstrap replicates | Sample frequency | runs/ chains | burnin | ASDSF^3^ | Bayes Factors^4^ /ML scores (-lLn) | ESS^4,5^ > 200 | PSRF^6^ |
| --- | --- | --- | --- | --- | --- | --- | --- | --- | --- | --- |
| RaxML | GTR G | 1 | 1000 | na | na | na | na | -23426.81 | na | na |
| RaxML | GTR G | **7** (by gene: 16S, 12S, COI, Cytb, ND6/ND4, H3A,18S) | 1000 | na | na | na | na | -22305.43 | na | na |
| RaxML | GTR G | **9** (16S+12S, COI1+Cytb1, COI2+Cytb2, COI3+Cytb3, ND6/ND4, H3A1, H3A2, H3A3, 18S) | 1000 | na | na | na | na | -21985.81 | na | na |
| RaxML | (GTR G)^8^ | **8** (16S+12S+Cytb1+Cytb2, COI1, COI2, COI3, Ctyb3, ND6/ND4, H3A1+H3A2, H3A3+18S)^8^ | 1000 | na | na | na | na | -22622.06 | na | na |
| Garli | TPM2uf G | 1 | 100 | na | na | na | na | -23373.57 | na | na |
| Garli | GTR G | 1 | 100 | na | na | na | na | -23359.42 | na | na |
| Garli | HKY G | 1 | 100 | na | na | na | na | -23030.02 | na | na |
| Garli | Mixed Models best (BIC)^7^ | **7** (by gene: 16S, 12S, COI, Cytb, ND6/ND4, H3A, 18S)^7^ | 100 | na | na | na | na | -23259.96 | na | na |
| Garli | Mixed Models  best (BIC)^8^ | **8** (16S+12S+Cytb1+Cytb2,  COI1, COI2, COI3, Ctyb3, ND6/ND4, H3A1+H3A2, H3A3+18S)^8^ | 100 | na | na | na | na | -23007.07 | na | na |
| MrBayes | GTR G | 1 | 17,000,000 | 1,000 | 4/4 | 10% | 0.002379 | -23492.78 | yes | 1 |
| MrBayes | HKY G | 1 | 17,000,000 | 1,000 | 4/4 | 10% | 0.002517 | -23497.31 | yes | 1 |
| Phycas | GTR G; polytomy prior | 1 | 500,000 | 100 | na | 20% | na | -23503.20 | na | na |
| BP | GTR G | 1 | 40,600,000 | 10,000 | 8/1 | 10% | na | -23502.21 | yes | na |
| BP | GTR G | 2 | 43,000,000 | 10,000 | 8/1 | 10% | na | -23332.23 | yes | na |
| BP | GTR G | 3 | 46,340,000 | 10,000 | 7/1 | 10% | na | -23291.97 | yes | na |

^1^ All others default; ^2^ number of partitions in bold font; different partitions separated by comma; ^3^ Average standard deviation of split frequencies; ^4^ estimated in Tracer v.1.5; ^5^ Effective Sample Size; ^6^ Potential Scale Reduction Factor for all parameters; ^7^ see Table 2; ^8^ PartitionFinder 1.0 (HKY+I+G;TrNef+G;F81;K81uf+G;TrN+G;TrN+I+G;JC+I;K80+G; suggested Best Model); BP BayesPhylogenies
