# Supplementary material for: Contrasting Phylogeography of Sandy vs. Rocky Supralittoral Isopods in the Megadiverse and Geologically Dynamic Gulf of California and Adjacent Areas
Source: PLoS One. 2013 Jul 2;8(7):e67827. doi: 10.1371/journal.pone.0067827 (PMC3699670; doi:10.1371/journal.pone.0067827)
Supplement: Table S5 — (DOCX) [file pone.0067827.s010.docx]

**Table S5**

Models, parameters, and priors used in the Maximum Likelihood and Bayesian phylogenetic analyses of the concatenated mitochondrial (MT) dataset.

| Method | Model and Priors^1^ | Partitioning scheme^2^ | iterations generations/bootstrap replicates | Sample frequency | runs/ chains | burnin | ASDSF^3^ | Bayes Factors^4^ /ML scores (-lLn) | ESS^4,5^ > 200 | PSRF^6^ |
| --- | --- | --- | --- | --- | --- | --- | --- | --- | --- | --- |
| RaxML | GTR G | 1 | 1000 | na | na | na | na | -19413.81 | na | na |
| Garli | TIM2 I G | 1 | 100 | na | na | na | na | -18935.40 | na | na |
| Garli | HKY G | 1 | 100 | na | na | na | na | -19262.02 | na | na |
| Garli | GTR G | 1 | 100 | na | na | na | na | -19833.38 | na | na |
| MrBayes | GTR G | 1 | 100,000,000 | 1,000 | 4/4 | 10% | 0.000781 | -19470.73 | yes | 1 |
| MrBayes | HKY G | 1 | 100,000,000 | 1,000 | 4/4 | 10% | 0.000936 | -19492.27 | yes | 1 |
| Phycas | GTR G; polytomy prior | 1 | 500,000 | 100 | na | 20% | na | -19485.60 | na | na |

^1^ All others default; ^2^ different partitions separated by comma; ^3^ Average standard deviation of split frequencies; ^4^ estimated in Tracer v.1.5; ^5^ Effective Sample Size; ^6^ Potential Scale Reduction Factor for all parameters.
