# Supplementary material for: Contrasting Phylogeography of Sandy vs. Rocky Supralittoral Isopods in the Megadiverse and Geologically Dynamic Gulf of California and Adjacent Areas
Source: PLoS One. 2013 Jul 2;8(7):e67827. doi: 10.1371/journal.pone.0067827 (PMC3699670; doi:10.1371/journal.pone.0067827)
Supplement: Table S6 — (DOCX) [file pone.0067827.s011.docx]

**Table S6**

Models, parameters, and priors used in the Maximum Likelihood and Bayesian phylogenetic analyses of the nuclear (NC) datasets.

| Gene | Method | Model and Priors^1^ | Partitioning scheme^2^ | iterations generations/bootstrap replicates | Sample frequency | runs/ chains | burnin | ASDSF^3^ | Bayes Factors^4^ /ML scores (-lLn) | ESS^4,5^ > 200 | PSRF^6^ |
| --- | --- | --- | --- | --- | --- | --- | --- | --- | --- | --- | --- |
| 18S rDNA | RaxML | GTR G | 1 | 1000 | na | na | na | na | -2565.73 | na | na |
|  | Garli | K80 G | 1 | 100 | na | na | na | na | -2593.56 | na | na |
|  | Garli | HKY G | 1 | 100 | na | na | na | na | -2581.50 | na | na |
|  | Garli | GTR G | 1 | 100 | na | na | na | na | -2409.38 | na | na |
|  | MrBayes | GTR G | 1 | 30,000,000 | 1,000 | 4/4 | 10% | 0.003538 | -2657.08 | yes | 1 |
|  | MrBayes | HKY G | 1 | 30,000,000 | 1,000 | 4/4 | 10% | 0.003554 | -2656.50 | yes | 1 |
|  | Phycas | GTR G; polytomy prior | 1 | 500,000 | 1,000 | na | 20% | na | -2644.56 | na | na |
| H3A | RaxML | GTR G | 1 | 1000 | na | na | na | na | -711.72 | na | na |
|  | RaxML | (GTR G)^7^ | 2 (by codon; H3A1+2, H3A3)^8^ | 1000 | na | na | na | na | -670.41 | na | na |
|  | Garli | TPM1 G | 1 | 100 | na | na | na | na | -648.94 | na | na |
|  | Garli | HKY G | 1 | 100 | na | na | na | na | -697.45 | na | na |
|  | Garli | GTR G | 1 | 100 | na | na | na | na | -691.27 | na | na |
|  | Garli | Mixed Model  best (BIC)^7^ | 2 (by codon; H3A1+2, H3A3)^8^ | 100 | na | na | na | na | -664.60 | na | na |
|  | MrBayes | GTR G | 1 | 30,000,000 | 1,000 | 4/4 | 10% | 0.002061 | 843.55 | yes | 1 |
|  | MrBayes | HKY G | 1 | 30,000,000 | 1,000 | 4/4 | 10% | 0.002243 | -845.62 | yes | 1 |
|  | Phycas | GTR G; polytomy prior |  | 500,000 | 1,000 | na | 20% | na | -782.60 | na | na |

^1^ All others default; ^2^ different partitions separated by comma; ^3^ Average standard deviation of split frequencies; ^4^ estimated in Tracer v.1.5; ^5^ Effective Sample Size; ^6^ Potential Scale Reduction Factor for all parameters; ^7^ PartitionFinder 1.0 (JC+I;K80; suggested Best Model); BP = BayesPhylogenies
