# Supplementary material for: Contrasting Phylogeography of Sandy vs. Rocky Supralittoral Isopods in the Megadiverse and Geologically Dynamic Gulf of California and Adjacent Areas
Source: PLoS One. 2013 Jul 2;8(7):e67827. doi: 10.1371/journal.pone.0067827 (PMC3699670; doi:10.1371/journal.pone.0067827)
Supplement: Table S7 — (DOCX) [file pone.0067827.s012.docx]

**Table S7**

Ranges of percent Kimura-2-parameter distances among the main *Tylos* clades found in the study area and the outgroup taxa. Lower matrix; COI gene distances. Upper matrix; the combined mitochondrial gene (16S rDNA+12S rDNA+Cytb+ND4/6) distance. Values on diagonal show maximum within-clade divergence (left: COI gene; right: the combined mitochondrial genes)

|  | 1 | 2 | A | B-I | B-II | C | D | | E | F | G | H | I |
| --- | --- | --- | --- | --- | --- | --- | --- | --- | --- | --- | --- | --- | --- |
| *T. niveus* (1) | **na** | 18.85 | 19.15-  21.06 | 19.72 | 16.29-  17.03 | 21.39 | | 26.65 | 17.67-  22.21 | 20.37-  23.15 | 16.14-  23.20 | 21.17-  21.80 | 21.66-  22.54 |
| Yaguanabo (2) | 15.60 | **na** | 20.65-  22.15 | 22.56 | 23.12-  23.24 | 23.43 | | 26.55 | 17.19-  24.15 | 22.16-  23.33 | 16.37-  24.98 | 22.14-  23.96 | 24.15-  24.59 |
| A (Pacific-CA) | 15.53-  15.93 | 16.62-  16.93 | **(0.59/0.72)** | 18.93-  19.52 | 18.20  19.07- | 19.10-  19.69 | | 20.98-  21.39 | 15.64-  22.07 | 19.59-  22.50 | 13.86-  21.22 | 18.73-  20.45 | 19.34-  20.72 |
| B-I (Mazatlan) | 13.64 | 17.20 | 12.22-  12.47 | **na** | 9.30-  9.89 | 18.61 | | 22.73 | 14.88-  21.48 | 20.00-  21.57 | 13.04-  19.75 | 18.82-  19.24 | 19.95-  20.44 |
| B-II (southern Mexico) | 15.29-  16.63 | 15.09-  15.40 | 12.22-  13.58 | 8.95-  9.07 | **(1.30/1.90)** | 18.01-18.86 | | 22.70-  23.16 | 14.72-  21.58 | 18.60-  20.93 | 13.51-  21.04 | 18.98-  20.65 | 20.13-  20.82 |
| C (Loreto) | 16.90 | 19.43 | 14.95-  15.36 | 12.50 | 13.18-  14.18 | **na** | | 21.21 | 15.27-  21.70 | 19.45-  20.38 | 14.17-  18.21 | 17.33-  18.68 | 17.78-  17.90 |
| D (Ceuta) | 16.06 | 19.06 | 11.43-  12.15 | 13.20 | 12.42-  12.93 | 13.71 | | **na** | 14.46-  20.47 | 18.78-  21.64 | 14.29-  22.00 | 20.01-  21.25 | 20.74-  21.11 |
| E | 16.95-  19.11 | 15.48-  18.69 | 14.00-  16.58 | 14.86-  15.22 | 13.66-  15.22 | 15.15-17.30 | | 12.07-  14.42 | **(7.66/5.90)** | 8.07-  16.99 | 12.65-  19.67 | 13.92-  18.95 | 14.32-  19.22 |
| F | 13.45-  14.71 | 16.36-  18.18 | 11.68-  15.62 | 10.22-  12.39 | 13.23-  14.57 | 12.60-14.34 | | 10.69-  11.51 | 11.14-  13.34 | **(6.86/7.40)** | 13.33-  20.00 | 16.84-  19.53 | 18.11-  20.14 |
| G (North) | 14.72-  16.64 | 16.66-  18.94 | 12.47-  14.65 | 12.52-  14.80 | 11.86-  14.31 | 12.70-  14.78 | | 10.93-  13.53 | 12.75-  17.02 | 11.09-  14.97 | **(6.08/4.86)** | 2.80-  4.87 | 2.79-  6.67 |
| H (South) | 16.74-  18.23 | 16.24-  18.23 | 13.18-  15.74 | 13.79-15.18 | 12.66-  14.48 | 13.02-  15.33 | | 11.94-  13.20 | 13.78-  16.93 | 12.16-  16.64 | 3.56-  6.76 | **(4.64/2.84)** | 5.07-  5.60 |
| I (Middle) | 16.43-  17.05 | 17.96-  18.28 | 13.45-  15.02 | 12.96-13.56 | 12.91-  13.83 | 13.77-  14.27 | | 13.15-  13.47 | 14.30-  16.32 | 11.98-  15.56 | 4.63-  7.05 | 4.24-  5.43 | **(0.54/0.90)** |
